# Exploratory Survey on European Consumer and Stakeholder Attitudes towards Alternatives for Surgical Castration of Piglets

**DOI:** 10.3390/ani10101758

**Published:** 2020-09-28

**Authors:** Marijke Aluwé, Evert Heyrman, João M. Almeida, Jakub Babol, Gianni Battacone, Jaroslav Čítek, Maria Font i Furnols, Andriy Getya, Danijel Karolyi, Eliza Kostyra, Kevin Kress, Goran Kušec, Daniel Mörlein, Anastasia Semenova, Martin Škrlep, Todor Stoyanchev, Igor Tomašević, Liliana Tudoreanu, Maren Van Son, Sylwia Żakowska-Biemans, Galia Zamaratskaia, Alice Van den Broeke, Macarena Egea

**Affiliations:** 1 Flanders Research Institute for Agriculture, Fisheries and Food (ILVO), Animal Sciences Unit, 9090 Melle, Belgium; evert.heyrman@ilvo.vlaanderen.be (E.H.); alice.vandenbroeke@ilvo.vlaanderen.be (A.V.d.B.); 2 Instituto Nacional de Investigação Agrária e Veterinária (INIAV), Quinta da Fonte Boa, 2005-048 Vale de Santarém, Portugal; joaoalmeida@iniav.pt; 3 Department of Biomedical Science and Veterinary Public Health, Swedish University of Agricultural Sciences, Box 7015, 750 07 Uppsala, Sweden; jakub.babol@slu.se; 4 Department of Agriculture, University of Sassari, Viale Italia 39, 07100 Sassari, Italy; battacon@uniss.it; 5 Department of Animal Science, Faculty of Agrobiology, Food and Natural Resources, Czech University of Life Sciences Prague (CZU), Kamycka 129, 16500 Prague, Czech Republic; citek@af.czu.cz; 6 Institute for Food and Agriculture Research and Technology (IRTA), Product Quality Program, Finca Camps i Armet, 17121 Monells, Girona, Spain; maria.font@irta.cat; 7 Animal Breeding Department, National University of Life and Environmental Sciences of Ukraine (NULES), Henerala Rodimtseva 19, 03041 Kyiv, Ukraine; getya@ukr.net; 8 Department of Animal Science, Faculty of Agriculture (UNIZG), University of Zagreb, Svetosimunska cesta 25, 10 000 Zagreb, Croatia; dkarolyi@agr.hr; 9 Institute of Human Nutrition Sciences, Warsaw University of Life Sciences (WULS-SGGW), ul. Nowoursynowska 159c, 02-787 Warsaw, Poland; eliza_kostyra@sggw.edu.pl (E.K.); sylwia_zakowska_biemans@sggw.edu.pl (S.Ż.-B.); 10 Department of Behavioral Physiology of Livestock, Institute of Animal Science, University of Hohenheim, Garbenstraße 17, 70599 Stuttgart, Germany; kress.kevin@uni-hohenheim.de; 11 Department of Animal Production and Biotechnology, Faculty of Agrobiotechnical Sciences Osijek, University of Osijek, Vladimira Preloga 1, 31000 Osijek, Croatia; gkusec@fazos.hr; 12 Department of Animal Sciences, University of Göttingen, Albrecht-Thaer-Weg 3, 37075 Göttingen, Germany; daniel.moerlein@uni-goettingen.de; 13 V.M. Gorbatov Federal Research Center for Food Systems of Russian Academy of Sciences, 26, Talalikhina str., 109316 Moscow, Russia; a.semenova@fncps.ru; 14 Agricultural Institute of Slovenia, Hacquetova ulica 17, SI-1000 Ljubljana, Slovenia; martin.skrlep@kis.si; 15 Department of Food safety and control of foodstuffs animal origin, Faculty of Veterinary medicine, Trakia University, Students Campus 6000, 6000 Stara Zagora, Bulgaria; todor.stoyanchev@uni-sz.bg; 16 Department of Animal Source Food Technology, Faculty of Agriculture, University of Belgrade, Nemanjina 6, 11080 Belgrade, Serbia; tbigor@agrif.bg.ac.rs; 17 Interdisciplinary Laboratory for Research on Heavy Metals Accumulation in the Food Chain and Modeling, Veterinary Medicine, Faculty of Veterinary Medicine and University of Agronomic Sciences, 011464 Bucharest, Romania; liliana_tudoreanu223@hotmail.co.uk; 18 Norsvin SA, Storhamargata 44, 2317 Hamar, Norway; maren.van.son@norsvin.no; 19 Department of Molecular Sciences, Swedish University of Agricultural Sciences, Box 7015, 750 07 Uppsala, Sweden; Galia.zamaratskaia@slu.se; 20 Department of Food Science and Technology, Veterinary Faculty, University of Murcia, 30071 Espinardo Murcia, Spain; macarena.egea@um.es

**Keywords:** acceptability, boar production, immunocastration, anaesthesia, analgesia, online questionnaire, cluster analysis, infographic

## Abstract

**Simple Summary:**

In many countries, surgical castration of piglets without pain relief or anaesthesia is still common practice. Castration is performed to minimise the incidence of boar taint, a bad taste (urine/fecal like), typically present in the meat of 5 to 10% of uncastrated male pigs. It also helps to avoid aggressive and sexual behaviour. For animal welfare reasons, alternatives are being considered, and in some countries, an alternative is already practiced. One option is to perform surgical castration with anaesthesia and relieve pain. A second option is to produce male pigs without castration, which requires detection of tainted carcasses in the slaughter house. A third option is to apply immunocastration: by a two-fold injection of a vaccine, the testes function is inhibited, which reduces boar-like behaviour and avoids boar taint. In this study, we evaluated the acceptability of each of these methods in 16 countries in Europe. Of the 4 presented options, the practice of surgical castration was least accepted (32%), whilst there was a high acceptance of castration with anaesthesia (85%), followed by immunocastration (71%) and production of boars (49%). The developed questionnaire and infographic can be used in future studies to further gain insights in consumer and stakeholder attitudes on this topic.

**Abstract:**

Surgical castration of piglets without pain relief is still common practice in many countries. Possible alternatives for surgical castration are application of pain relief or anaesthesia or production of boars (entire males) and immunocastrates. Each of these alternatives faces advantages and disadvantages which may result in different citizen attitudes and consumers acceptability. Understanding which practice is acceptable to whom and why may further stimulate implementation. Consumer (*n* = 3251) and stakeholder (*n* = 1027) attitudes towards surgical castration without pain relief, surgical castration with anaesthesia, immunocastration, and production of boars were surveyed from April to June 2020 via an online questionnaire in 16 countries (>175 respondents per country). Surgical castration without pain relief was separated from each of the alternatives due to animal welfare and showed the lowest acceptability (32%). Within the alternatives, a further partitioning between the alternatives was based on perceived quality and food safety, with an acceptance of 85% for applying anaesthesia, 71% for immunocastration, and 49% for boar production. Differences depending on professional involvement and familiarity with agriculture could be observed, mainly for the acceptance of surgical castration without anaesthesia, immunocastration, and boars. Castration with anaesthesia was highly accepted by all types of respondents.

## 1. Introduction

Surgical castration of male piglets is applied to eliminate the risk of boar taint and to manage typical boar behaviour. This procedure is painful when applied without pain relief (referred to as “castration” below), which is a common practice in many countries. This practice has been strongly contested by animal welfare organizations in several European countries. Citizens and pork production chain as well as other societal stakeholders have demanded a ban on surgical castration without pain relief and the European Commission set a deadline of 2018 [1,2]. Possible alternatives are the application of pain relief or anaesthesia, the use of immunocastration, or pork production with boars (entire male pigs). However, while alternatives are applied in a number of countries, others have not adhered to this deadline. The production system of male pigs currently differs between and even within countries.

**Figure 1:**
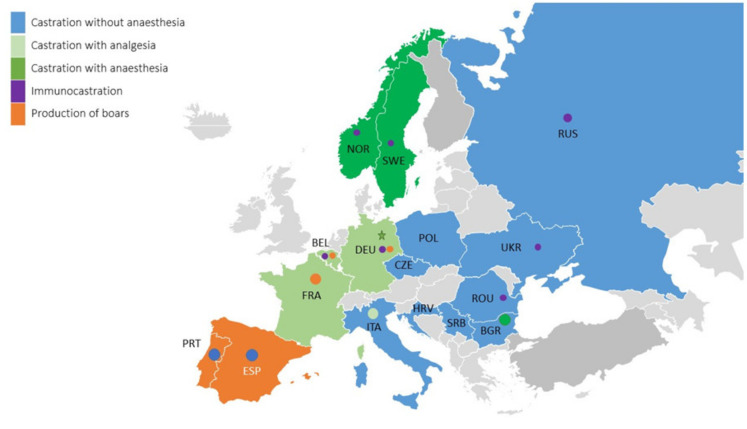
Currently applied production methods of male pigs for the countries included in this survey. Background colour indicates the main male pig production method and the dot indicates the additionally applied methods, whilst a star indicates the method as foreseen for implementation as main method in 2021 (source: based on [4], updated by the European Cooperation in Science and Technology (COST) consortium).

Application of analgesia is demanded by several quality assurance programs, e.g., in Belgium, Germany, and France (Figure 1). Some countries have a longer tradition of using anaesthesia during castration, such as Norway (by the veterinarian), Sweden (by the farmer), and Switzerland (farmer/vet), while this practice was recently introduced in Denmark (2020, by the farmer) and will be introduced in Germany (2021 [3,4,5,6], farmer). In Bulgaria, castration with anaesthesia is applied by the vet at higher weights for home grown pigs. Production of boars is common in Spain, Portugal, Ireland, and United Kingdom, and since 2010, introduced in countries like the Netherlands, Belgium, and France. Immunocastration is applied to 5 up to 10% of the male piglets in a number of countries as well [4,7].

In the recent past, surgical castration with application of analgesia or anaesthesia have been regarded as either a short-term solution or only applicable to specific pork production systems such as artisanally produced, high-quality pork products, or organic pig production. This practice faces challenges, such as the legal authorization for applying anaesthesia by the vet or farmer after training, and correct implementation in practice. Alternatives such as immunocastration or production of boars are practiced to some extent in a number of countries. However, these practices have not taken root in many countries for different reasons. For boar production, disadvantages are mainly related to pork quality, with boar taint as main issue. Boar taint is found in 5–10% of entire males, but prevalence could also be higher depending on detection method, thresholds used and the pig’s characteristics (e.g., genetic, age at slaughter), and environmental (e.g., dietary, management) factors [8,9,10]. Carcasses with boar taint should therefore be detected at the slaughter line, but an objective (instrumental) online detection method has longtime not been available. Progress is being made, particularly with the introduction of a new method in Denmark [11,12]. There are other issues, however: boars show poorer results for intramuscular fat and water-holding capacity, which may also result in lower sensory quality [13,14,15,16]. Immunocastration can be applied, since the vaccine Improvac (Zoetis, Louvain-la-Neuve, Belgium) received EU marketing authorization approval in 2009. Application of immunocastration can solve a major part of the quality issues related to the production of boars as it prevents boar taint and shifts the meat quality more towards that of castrated male pigs [14,17,18,19]. However, large-scale implementation of immunocastration is being held back mainly because consumer acceptance of this practice is being questioned by stakeholders in the pork chain. Insights in the level of acceptance and a better understanding of which alternative is acceptable to whom and why may increase insights and stimulate implementation of the alternatives.

Different methodologies are used to evaluate consumer acceptability. Perception of piglet castration is measured via qualitative (e.g., focus group discussions) [20,21,22] and quantitative studies such as choice experiments [23], ranking one or more alternatives for surgical castration, and evaluating the acceptability [24,25] or assessing their willingness to pay for meat and meat products [26,27] via face-to-face or online surveys [28]. In each of the methodologies, the lack of knowledge about pig production as well as the topic of surgical castration and its alternatives is important to take into account when studying consumer attitudes [29]. With some variation between studies, 40 to 50% of the consumers indicated awareness of castration of male pigs [21,25,27,28], while only 14 to 21% indicated that they were well aware of this practice [25]. Knowledge on this topic also strongly differs (18 to 57%) depending on factors such as background (rural/urban) and age (younger people are also less aware) [21,28]. While animal behaviour and meat quality are mentioned as reasons for castration, only few people mention the term “boar taint” in focus groups [21,29]. This also implies that information needs to be provided when studying attitudes in this field. The information provided clearly differs between studies in terms of terminology used, extensiveness, the pros and cons of the various alternatives, and the format, all of which might affect the results [21,22,30]. Besides the dissimilarities in study approach and background information, consumer characteristics may also differ between studies of only one country, as well as between consumers. Several studies stipulate the heterogeneity in consumer attitudes toward castration and alternatives for castration [25,31]. Besides the known differences between consumers in sensory sensitivity and/or preference to the boar taint compounds and most importantly androstenone, consumer characteristics and the importance of the different attributes differ. Attitudes and expectations are mainly influenced by information about process characteristics, beliefs, and feelings, but the final consumer decision-making also involves the taste of the meat, whether it is healthy or not and the convenience of purchase and preparation. It is assumed that consumers consider a trade-off between animal welfare, food quality, and food safety when purchasing pork [23]. The study of Heid and Hamm [22] indicates that for alternatives with surgical castration (anaesthesia, analgesia), animal welfare aspects play the main role in willingness to pay, while in the case of immunocastration, food safety is more important. For production of boars, taste and to a lesser extent animal welfare were considered decisive. Differences in citizen attitudes and consumer acceptability of the alternatives depending on the individual importance of animal welfare, taste, and food safety can be explored using cluster analysis [25].

Overall, the number of studies that evaluated consumer acceptance of the different alternatives in Europe or covering multiple countries is limited and recent studies are lacking [25]. It can also be expected that attitudes differ between countries [31]. Therefore, the main aims of the study were twofold: (1) to evaluate consumers attitudes towards alternatives for surgical castration of piglets in Western and Eastern European countries with a focus on perception of animal welfare, taste, and food safety based on the background information provided to the consumers, and (2) to profile respondents based on socio-demographics as well as their involvement and specific role in the pig production chain.

## 2. Materials and Methods

### 2.1. Research Approach and Sampling

Consumer attitudes towards alternatives for surgical castration of pigs were collected via an internet survey (LimeSurvey) from April to July 2020. The questionnaire was made available in 20 languages (Bulgarian, Catalan, Croatian, Czech, Dutch, French, German, Italian, Macedonian, Norwegian, Polish, Portuguese, Romanian, Serbian, Slovenian, Spanish, Swedish, and Ukranian) and covered 23 countries i.e., Belgium (BEL), Bulgaria (BGR), Czechia (CZE), Switzerland (CHE), Germany (DEU), Spain (ESP), France (FRA), Croatia (HRV), Italy (ITA), Netherlands (NLD), Norway (NOR), Poland (POL), Portugal (PRT), Romania (ROU), Russia (RUS), Serbia (SRB), Slovenia (SVN), Sweden (SWE) and Ukraine (UKR). Responses were collected by convenience sampling via social media and mail by the COST IPEMA network members and their networks and families. Participation in the survey was voluntary and completely anonymous, and data was handled according to GDPR guidelines. Only respondents consuming pork could participate in the survey. Finally, countries with more than 175 respondents were included in the final manuscript, representing 16 countries. A total of 4278 persons participated in the survey. Average answering time was around 11 min 50 s.

The questionnaire consisted of 6 parts, each presented on a separate page.

**Figure 2:**
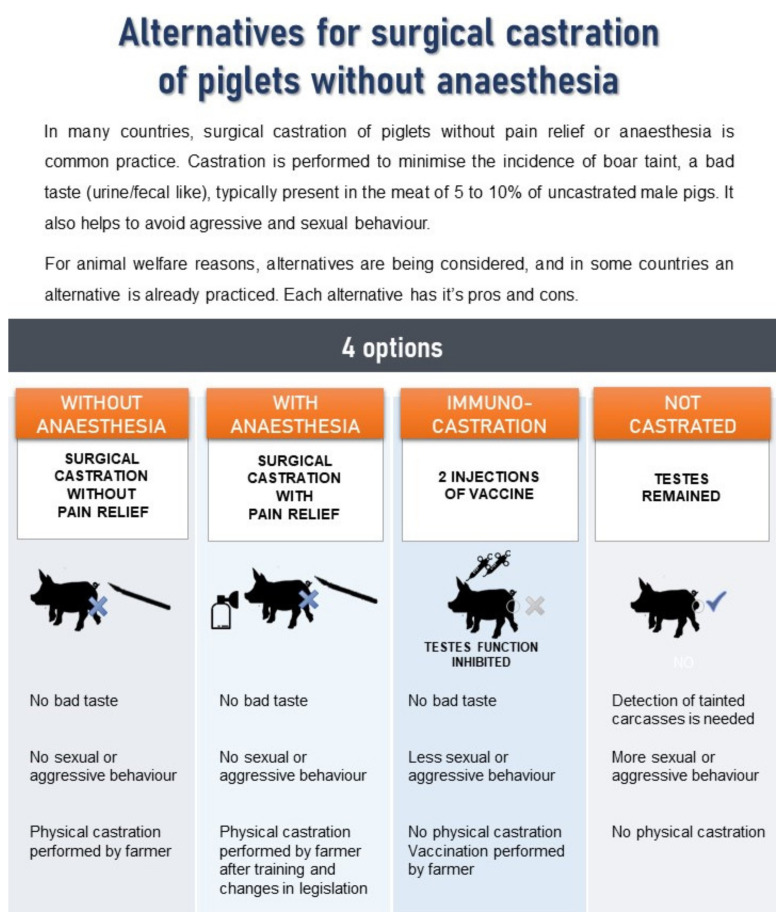
Infographic with information was provided before questioning the acceptance and attitude towards the different options.

Part 1 consisted of general questions regarding consumption of meat and specifically pork. Next, consumers were questioned about their pork liking, purchasing habits and drivers, followed by questions on their experience with bad odours and flavours and about their (potential) reaction when experiencing a bad odour/flavour whilst preparing meat. Finally, they were asked about their attitude towards vaccination in general. In part 2, respondents were asked about their awareness of piglet castration without anaesthesia as normal practice in many countries. In part 3, respondents were asked if they knew how most male pigs were produced in their country. In part 4, participants received basic information about each practice and reason of piglet castration followed by an explanation about the 4 options of how male pigs can be produced. We chose to present information in an infographic instead of only via text to enhance information capture by the consumer (Figure 2). In part 5, attitudes towards the 4 options were assessed in three ways. First, consumers were asked to score the acceptability of each option based on the study of Fredriksen et al. [21]. Possible answer options were: “totally acceptable”, “possibly acceptable”, “not acceptable”, and “I don’t know”. Second, consumers scored their agreement (score 1: totally disagree to 7: totally agree) towards 5 statements: “If the castration method was clearly labelled, I would surely buy…” (*WTB*), “I think that it is totally safe to eat meat from …” (*SAFE*), “When I think about the following production method, it seems logical to me that animal welfare is best for …” (*WELFARE*), “If I consider the tastiness of meat, I prefer meat from…” (*TASTE*), and ”I am completely convinced that this is the best option …” (“*BEST PRACTICE*”). Third, respondents were asked to check all terms that apply (CATA) to ideal pork (production) and the 4 options. Terms were based on 7 positive attributes (good quality, natural, safe to eat, free of pain, welfare friendly, cheap, practically feasible) and 7 negative attributes (bad taste, residues, hormones, stressful, cruel practice, expensive, difficult to understand). In part 6, respondents were asked to fill in socio-demographic information (country, age category, gender, living area, education level). Additionally, their professional and personal involvement in animal production was surveyed to enable analysis of stakeholder attitudes.

The questionnaire was first developed based on a literature review and existing questionnaires, and then further optimized in discussion with experts of the COST IPEMA network, taking the trade-off between animal welfare, food quality, and food safety into account. After a first online pretest with 30 respondents (Belgium, Spain), some first changes were made to improve phrasing. After a second pretest (45 questions, 16 min) with 152 respondents (Belgium, Spain), further improvements were made related to phrasing and simplification of the questions, reduction of the number of question and statements, and a reduction in the number of CATA terms. In a final step, the questionnaire was tested and discussed in a focus group (Belgium, 10 participants), primarily to optimize the description of the CATA terms.

### 2.2. Statistical Analysis

All data analysis was done in R [32]. For the CATA terms a correspondence analysis was performed using Hellinger distances, all alternatives were compared to the ideal product, and Cochran’s Q-test was used to find significant differences between alternatives within countries (*p* < 0.05) [33]. For the statement variables, a hierarchical cluster analysis was performed across countries using the Ward method and Euclidian distance. The number of clusters to retain was based on the percentage of within-cluster variance drop after adding an additional cluster. These same variables were also analyzed in a first analysis of variance with gender, age, education, area, and professional and non-professional involvement as fixed effects. A second and third analysis of variance used country and cluster as fixed effect, respectively.

Statement variables were further analyzed using analysis of variance using country and alternative as fixed effects and its interaction to find significant differences between alternatives within countries. Acceptance variables were summarized for each alternative over the different countries [4].

## 3. Results

### 3.1. Demographic Profile and Pork Consumption Characteristics

In total, 4278 respondents from 16 countries participated in the trial, with 177 to 506 participants per country (Table 1). Women were overrepresented, especially for BGR, FRA, NOR, POL, PRT, ROU (>60%) and SWE (>70%). All age categories were represented, with 18% being younger than 25, 34% between 25 and 39, 41% between 40 and 64, and 7% older than 64 years of age. Mainly, respondents of POL (>60%), ROU and UKR (>30%) were younger than 25, whilst the group of >64 year old respondents was highest for DEU (21%). Regarding living area, 35% indicated living in a big city, 29% in a medium-sized town or city, and 36% in a rural area or small town. The respondents from BGR and RUS were mainly situated in a big city, those from ESP and PRT in a medium–sized city, and those from BEL and ITA in a rural area or small town. The majority of the respondents had a university degree (Master’s degree equivalent) as their highest diploma (66%), especially for respondents of BGR, ESP, PRT, and SWE (>80%). Sixteen percent had a higher, non-university degree (Bachelor’s degree equivalent) (>30% for BEL) and 18% had a primary or secondary school diploma (>40% for DEU). Professional involvement was 24% and was closely linked to pig production (71%), with 37% working as a researcher, 24% veterinarian, 18% farmer, 14% processing sector, 12% supply chain, and 7% butcher or retail. Professional involvement was highest for HRV, CZE, ESP, FRA, and PRT (>30%). Familiarity with pig production (i.e., non-professional involvement) was highest for BEL, BGR, and ROU (>30%) “regular contact with animal production”, and for UKR “grew up on a farm” (>30%). Respondents of DEU, POL, and RUS were least familiar with pig production.

### 3.2. Pork Consumption Characteristics, Background Knowledge, and Experience

Overall, most respondents indicated that they consume pork 1 to 2 times per week (36%) or 3 to 4 times per week (29%), whilst 18% consume pork less than once per week, and 17% more than 4 times per week (Table 2). Liking of pork chops as well as minced meat products was overall 5.6 and 5.5, corresponding with slight to moderate liking. Pork is mainly bought in the supermarket (66%) and at the butcher (44%), with strikingly less butcher purchasing in NOR (2%) and SWE (8%). Good taste (86%) and guaranteed food safety (84%) were considered as most important attributes at purchase; this was consistent for all countries. Second most important were high tenderness (60%), produced locally (59%) and better animal welfare (55%), but the importance of these attributes varied greatly between countries. Lowest price (24%) was least frequently considered as most important attribute.

Overall, 72% of the respondents were confident that the meat they eat is safe. The highest level was found for BEL, ESP, PRT, and NOR (>80%), whilst this level was less than 60% for BGR, ITA, RUS, and UKR. Twenty percent of the respondents had a negative attitude towards the use of vaccines in general. Respondents of ESP, PRT, SWE, and NOR were in general more positive towards the use of vaccines (negative attitude <10%), whilst respondents from CZE, RUS, and SRB were more negative (negative attitude >30%).

Overall, 59% of the respondents indicated to be aware of versus 41% unaware of the practice of piglet castration. When asked about how male pigs are mainly produced, 56% of the respondents indicated that they did not know. This segment was highest (>65%) for ITA, POL, ROU, RUS, and SRB. In CZE, FRA, and SWE, most of the respondents were able to indicate the correct alternative. The application of anaesthesia or analgesia as well as the application of immunocastration were generally overrated as main production method. Around 30% of the respondents indicated to have experienced a bad smell or taste when consuming pork. When respondents were asked to indicate what they would do if they experience a bad odour or taste in a piece of meat, most of them indicated that they would complain to the shop, in person (32%) or via email (8%), or that they would no longer buy pork in that specific shop (33%). Another 20% of the respondent would just give it another chance. A minority indicated to stop eating that type of pork (4%) or post negative comments in social media (2%).

In order to evaluate the different options, background information was provided to the respondents by means of an infographic. Overall, 77% of the respondents indicated that the provided information was sufficient, and 56% indicated that they would like to learn more (independent of whether they found the information to be sufficient).

### 3.3. Overall Acceptability of Alternatives to Surgical Castration

**Figure 3:**
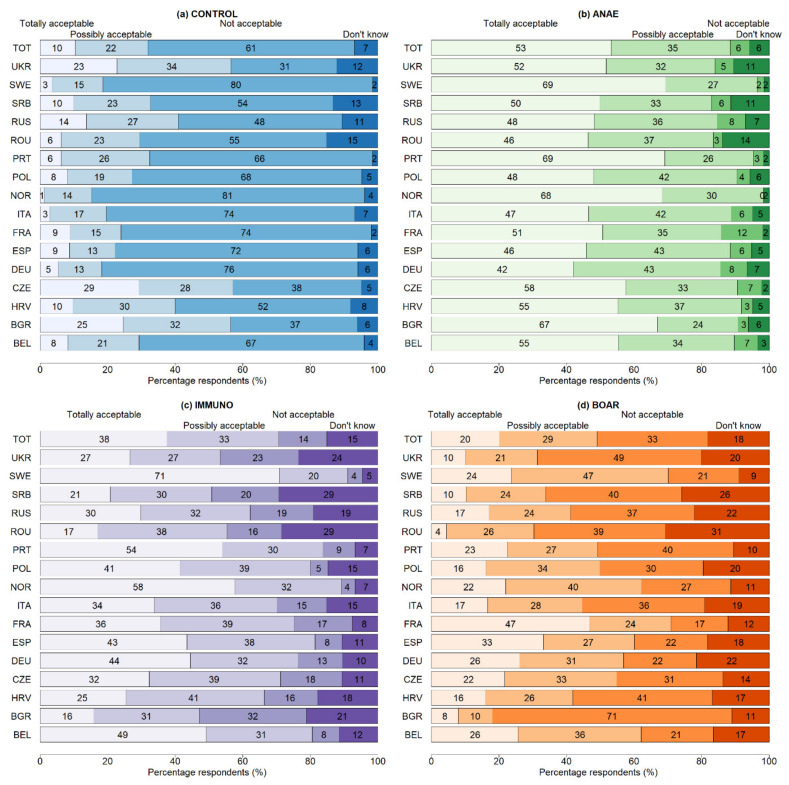
Acceptability of castration alternatives per country for (**a**) Castration without pain relief—CONTROL, (**b**) castration with anaesthesia—ANAE, (**c**) Immunocastration—IMMUNO, (**d**) no castration—BOAR. TOT—total sample.

Respondents rated the different alternatives after they received the infographic with background information. The overall percentage of totally acceptable was 53% for castration with anaesthesia (ANAE), 38% for immunocastration (IMMUNO), 20% for production of boars (BOAR), and 10% for castration without anaesthesia (CONTROL) (Figure 3). In contrast, 61% indicated CONTROL as not acceptable, 33% for BOAR, 14% for IMMUNO, and 6% for ANAE. In general, the acceptability was thus highest for ANAE, in particular for respondents of SWE, NOR, PRT, and BGR. Mainly respondents from SWE, PRT, NOR, and BEL were in favour (>49%) of IMMUNO, in contrast to respondents of BGR, SRB, and UKR (>20%). Respondents of FRA and ESP accepted BOAR most often (>33%) and the share of non-acceptance of BOAR was also lower for respondents of BEL and SWE. Respondents of BGR were least accepting BOAR. Overall, acceptance was lowest for CONTROL, especially in DEU, ESP, FRA, ITA, NOR, and SWE (>72%). Although pro CONTROL levels were low overall, respondents from UKR, CZE, and BGR scored highest (>23% totally acceptable).

### 3.4. Perception of Key Features Related to Pig Production and Meat: Evaluation and Correspondence Analysis

**Figure 4:**
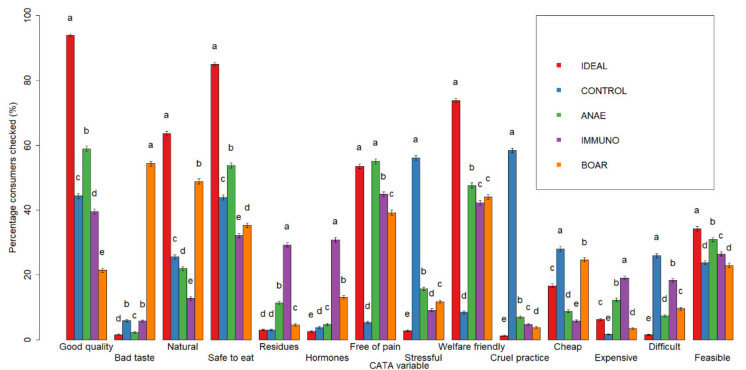
Frequence of Check-All-That-Apply (CATA) scoring of the ideal piece of pork (IDEAL), castration without pain relief (CONTROL), castration with anaesthesia (ANAE), immunocastration (IMMUNO), and no castration (BOAR). ^a–e^ different superscripts indicate significant differences between alternatives per CATA item (*p* < 0.05).

All 14 CATA terms were selected frequently by the respondents (>20%) for at least one of the alternatives, with the exception of “expensive” (Figure 4). Mainly “good quality”, “safe to eat”, “welfare friendly”, and “natural” were more frequently checked for IDEAL compared to each of the options (>60%). CONTROL had the highest frequency of “cruel practice”, “stressful”, and in a lesser extent also “difficult” and “cheap”. ANAE showed the highest frequency of “safe to eat”, “free of pain”, and “welfare friendly”. IMMUNO was mainly associated with higher frequencies for “welfare friendly”, and in a lesser extent also to “hormones”, “residues”, and “difficult”. BOAR was most associated with higher frequencies of “bad taste”, but also “natural”, “welfare friendly”, and “cheap”.

**Figure 5:**
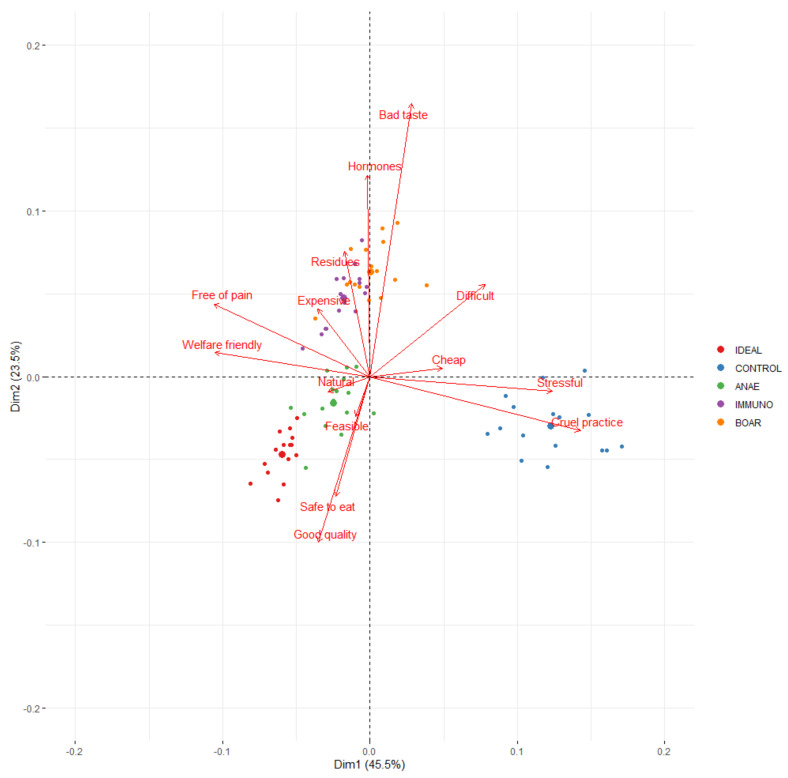
Correspondence analysis of CATA variables plotted for each alternative for the ideal piece of pork (IDEAL), castration without pain relief (CONTROL), castration with anaesthesia (ANAE), immunocastration (IMMUNO), and no castration (BOAR) with the small dots representing the results of a country per option and the large dot representing the average of each out the five options.

The same associations were observed within the correspondence analysis (Figure 5). The first axis (46%) differentiates CONTROL from the other three options and IDEAL based mainly on the terms related to animal welfare, i.e., “cruel practice”, “stressful” versus “welfare friendly” and “free of pain”. IDEAL is strongly associated with all the positive welfare characteristics and CONTROL with the negative ones, whilst the three alternatives are also more related to the positive welfare characteristics. The second axis (24%) allows further differentiation between IDEAL and the alternatives. IDEAL is mainly linked with “good quality” and “safe to eat” vs. BOAR opposite primarily due to its association with bad taste. IMMUNO is also, but less strongly, positioned on the same side as BOAR due to its association with “hormones”. Within the 4 options, ANAE and CONTROL are situated at the same side as IDEAL, but close to the origin, indicating that “food quality” and “food safety” are less determinant for these options.

### 3.5. Perception of the Alternatives Based on Different Statements

**Figure 6:**
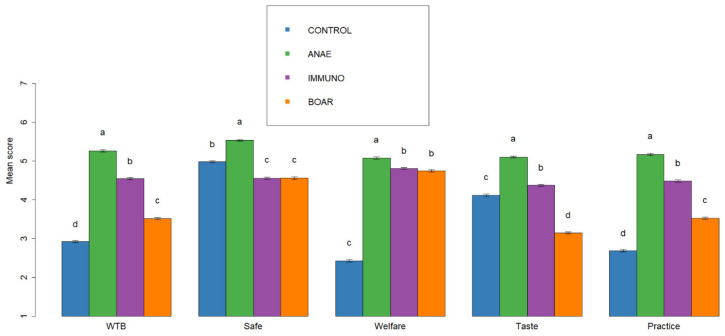
Overall level of agreement scored on a scale from 1 (totally disagree) to 7 (totally agree) towards different statements for the different castration alternatives “If the castration method was clearly labelled, I would surely buy…” (WTB), “I think that is totally safe to eat meat from …” (SAFE), “When I think about the following production method, it seems logical to me that animal welfare is best for …” (WELFARE), “If I consider the tastiness of meat, I prefer meat from…” (TASTE), ”I am completely convinced that this is the best option …” (BEST PRACTICE) for castration without pain relief—CONTROL, castration with anaesthesia—ANAE, Immunocastration — IMMUNO, and no castration—BOAR. ^a–d^ different superscripts indicate significant differences within statement variables per option (*p* < 0.05).

Overall, respondents agreed most with ANAE followed by IMMUNO and disagreed with BOAR, and disagreed especially strongly with CONTROL for *BEST PRACTICE* (“I am completely convinced that this is the best option“) and *WTB* (“If the castration method was clearly labelled, I would surely buy”) (Figure 6). For ANAE, there was most agreement with the statements regarding *SAFE* (“totally safe to eat”), *WELFARE* (“animal welfare is best”), and *TASTE* (“If I consider the tastiness of the meat, I prefer meat from”) compared to the other options. For BOAR and IMMUNO, level of agreement was similar for *SAFE*, but both lower than CONTROL and ANAE. BOAR and IMMUNO scored slightly lower for *WELFARE* compared to ANAE, but all much better than CONTROL. For *TASTE*, respondents agreed most with ANAE, followed by IMMUNO and CONTROL; respondents disagreed most frequently with BOAR.

**Figure 7:**
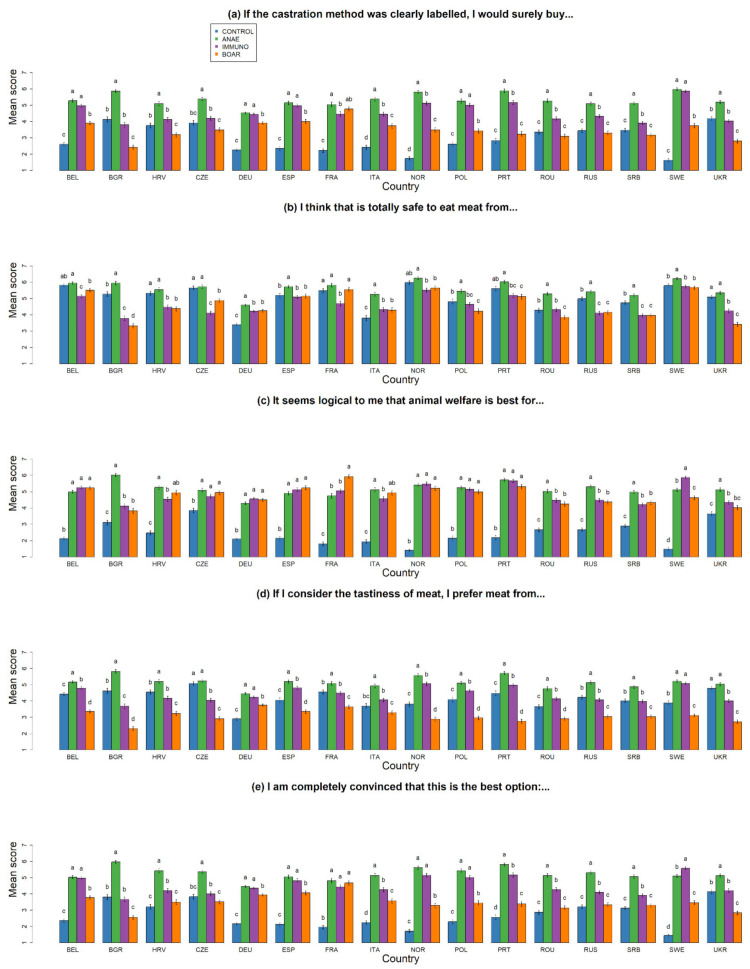
Overall level of agreement towards different statements for the different castration alternatives per country (**a**) “If the castration method was clearly labelled, I would surely buy…” (WTB), (**b**) “I think that is totally safe to eat meat from …” (SAFE), (**c**) “When I think about the following production method, it seems logical to me that animal welfare is best for …” (WELFARE), (**d**) “If I consider the tastiness of meat, I prefer meat from…” (TASTE), (**e**) ”I am completely convinced that this is the best option …” (BEST PRACTICE) for castration without pain relief—CONTROL, castration with anaesthesia—ANAE, Immunocastration—IMMUNO, and no castration—BOAR. ^a–d^ different superscripts indicate significant differences within countries per option (*p* < 0.05).

Some country differences could again be observed here (Figure 7). In BEL, DEU, ESP, POL, and SWE, respondents agreed equally with the *WTB* statement for IMMUNO and ANAE. For DEU, ESP, POL, SWE, as well as ITA and ROU, agreement with *SAFE* was rated either higher or equal for IMMUNO and CONTROL. In FRA, agreement for *WTB* was also high for BOAR and ANAE. This is also visible in the higher agreement score for *WELFARE* compared to the other options and equal score for *BEST PRACTICE* as ANAE and IMMUNO. In contrast, respondents of BGR, HRV, UKR, and respondents of CZE, PRT, ROU, RUS, and SRB showed more or equal disagreement with *WTB* for BOAR as compared to ANAE. This is reflected in a lower agreement with *SAFE* for BOAR compared to CONTROL in BGR, HRV, PRT, ROU, RUS, SRB, UKR, and POL. Respondents of UKR also agreed less with *WELFARE* for BOAR.

### 3.6. Effect of Demographics and (Non-)Professional Involvement

Male respondents agreed more with the practice of CONTROL (for all statements) and agreed more with BOAR on the *SAFE* and *WELFARE* statements compared to female respondents (Table 3). Female respondents assigned a slightly higher score to ANAE and IMMUNO for *WTB* and *WELFARE*. The main difference was in acceptability of CONTROL, with 38% acceptance by male and 27% by female respondents. Some differences could be observed between the different age groups, but these are generally small. The younger group of respondents (<25 years of age) gave a higher score to CONTROL and IMMUNO for *WTB* and *WELFARE* compared to the >25 and >40 years of age groups. The younger group scored *SAFE* and *WELFARE* as lower for BOAR. Again, mainly the acceptability of CONTROL was different, with 38% acceptance by the youngest respondents and 27% by the oldest group. In general, living area did not affect agreement scores (data not shown). For each of the statements, ANAE and IMMUNO received a higher score by the respondents with a university degree. The *SAFE* and *WELFARE* statements were also scored higher by this group for BOAR, whilst *TASTE* received a lower score. For CONTROL as well, *SAFE* and *TASTE* received a higher score by this group, whilst *WELFARE* scored slightly lower. These higher scores are also reflected in higher acceptance of all options.

Professionally-involved respondents always gave a higher score to each of the statements for CONTROL, ANAE, and IMMUNO than people who were not professionally involved. Only for BOAR, non-professionals indicated a slightly higher agreement with the statements regarding *WTB* and *TASTE*. Acceptance of CONTROL was higher for professionally-involved respondents (48% vs. 27%), with the highest acceptance by the farmers (61%) (Figure S1). ANAE was highly accepted by the not involved respondents (87%) and even more by the professional respondents (89–95%). Only farmers showed a slightly lower acceptance (83%). IMMUNO was more accepted by the professionally-involved respondents (78% versus 68%), and varied from 70% for farmers and processing up to 86% for veterinarians. For BOAR, highest variation was noted among professionally-involved stakeholders. Researchers showed the highest acceptability (63%), and processors the lowest (29%), whilst the other respondents were intermediate (43–52%).

Regarding impact of familiarity with agriculture, trends are comparable to professional involvement. Respondents with no or limited familiarity with agriculture agree less with all aspects of CONTROL and ANAE to a lesser extent. For IMMUNO, numerical differences are small, but are generally lower for the respondents that grew up on a farm. For BOAR, *WTB* and *TASTE* was lower for respondents that grew up on a farm versus respondents who are not familiar and intermediate for those with regular contact.

### 3.7. Cluster Analysis

Three clusters could be defined based on the respondents’ agreement to the different statement for each of the alternatives, representing 45% (*n* = 1910), 38% (*n* = 1619), and 18% (*n* = 749) of all respondents, respectively (Table 4).

Cluster 1 has the highest share of females (60% vs. 38% male), is highly familiar with agriculture (41%), and most professionally involved (31%). This cluster is well aware of piglet castration (69%), has the most positive attitude towards the use of vaccines (88%), and a high confidence in food safety (79%). Good taste and tenderness and animal welfare were more outspoken as important purchase attributes. Respondents of SWE, NOR, BEL, ESP, and PRT are more than averagely represented (>50%). Cluster 2 contains the least professionally-involved (23%) and agriculture familiar respondents (32%) and they are least aware of piglet castration (45%). This cluster is relatively less positive about the use of vaccines (73%), least confident in the food safety (63%), and has the least heavy pork consumers (10% > 4 times per week). Organically produced (44%) and low-fat content (36%) are more outspoken as purchase attributes. Respondents of DEU are most represented (61%). Cluster 3 has a higher share of male respondents (44% vs. 54% female), is most familiar with agriculture (54%), and moderately professionally involved (27%). This cluster is well aware of piglet castration (64%), less positive about the use of vaccines (73%), and moderately confident in food safety (71%). Good taste and tenderness, local production, and food safety are more outspoken as purchase attributes. This cluster contains the highest amount of pork consumers (16% > 4 times per week) and is more represented by BGR respondents (40%).

Cluster 1 can be considered as for ANAE and IMMUNO, and against CONTROL (Table 4, Figure S2). They disagree with the practice of CONTROL (*WTB*: 2.8) for animal welfare reasons, but consider the meat as safe and have no outspoken position regarding taste. IMMUNO (*WTB*: 5.8) and ANAE (*WTB*: 5.6) are considered as valid alternatives for CONTROL based on safety, welfare, and taste. BOAR is less favored (*WTB*: 3.5), due to a perception of it being less tasty, although this alternative is considered safe, animal welfare friendly, and natural. Cluster 2 also disagrees with CONTROL (*WTB*: 2.5) because of animal welfare, but considers it safe and tasty. This cluster has no outspoken opinion about the three alternatives, however. Cluster 3 has a more neutral attitude towards CONTROL (*WTB*:3.6) based on a positive score for safety and taste, and a negative score for animal welfare. They consider ANAE as good alternative (*WTB*: 6.3) based on welfare, taste, and safety, but are against IMMUNO (*WTB*:2.9) and BOAR (*WTB*:2.8). They reject IMMUNO for reasons of safety and taste and to a lesser extent also welfare. They disagree with BOAR for reasons of taste and to a lesser extent also safety.

A similar cluster analysis was performed for all respondents that are not professionally involved (data not shown). The three clusters were defined by comparable characteristics, with 42% of the respondents in cluster 1, 35% in cluster 2, and 24% in cluster 3. In general, it can be stated that the attitude of these non-professionally involved respondents is more outspoken regarding CONTROL and more neutral regarding IMMUNO. The main differences were present in clusters 1 and 3, which were also the most professionally involved for the overall dataset. For the non-professional respondents, cluster 1 was again for ANAE (*WTB*: 5.9) and IMMUNO (*WTB*: 5.4) and more outspokenly against CONTROL (*WTB*: 1.9). Cluster 2 was against CONTROL (*WTB*: 2.7) and neutral regarding the alternatives. Cluster 3 had a slightly more positive attitude towards CONTROL (*WTB*: 4.3) and towards ANAE (*WTB*: 5.8), was against BOAR (*WTB*: 2.9), and more neutral towards IMMUNO (*WTB*: 3.7).

## 4. Discussion

### 4.1. General Characteristics, Involvement, Awareness, and Background Information

Respondents were recruited via convenience sampling with a special emphasis to include professionally-involved stakeholders. As a result, respondents are characterized by a high professional involvement (24%), high familiarity with agriculture (40%), and are highly educated (66% university degree). Female respondents are slightly overrepresented (58 vs. 40%), whilst the different age categories were well represented overall, although this distribution also differs between countries and the share of +64 years old is rather on the lower side. The results of this survey are therefore mainly exploratory and do not allow strong conclusions to be made between countries. The study design does enable evaluation of the effect of different respondent characteristics and degree and type of involvement on the attitude towards surgical castration and the different alternatives. The developed questionnaire and infographic can be used in future studies.

Due to the specific respondent characteristics, the overall awareness of piglet castration within the entire group of respondents (59%) was higher than reported in other studies. Only 50% of the respondents that were not professionally involved were aware of piglet castration vs. 86% of the professionally involved respondents. When respondents were asked to indicate if they knew which method was practiced in their country, the numbers were lower (43%) and not all respondents were able to select the correct answer. An awareness of 40 to 50% of piglet castration was also reported in the literature [21,25,27,28], while only 14 to 21% reported being well aware of it [25]. Within the current study, no specific questions regarding awareness of the alternatives have been asked. In a previous study of Vanhonacker and Verbeke [25], about 10% of the respondents said they were familiar with the concept of boar taint, while only 1% was familiar with immunocastration. In this study, depending on the country, 24 to 44% of the respondents indicated to have experienced a bad smell or taste when consuming pork. This variation was not specifically related to countries that produce entire male pigs, however, indicating that a bad smell or taste may be related to other aspects than boar taint. It can be concluded that non-professionally involved respondents generally answered based on the information that was presented in the infographic and much less on existing knowledge. At the end of the questionnaire, respondents were asked if they thought they received sufficient information to answer all questions properly. The majority of the respondents (77%) indicated that the information provided was sufficient. Providing background information in a comprehensive and objective manner is a challenging task. Immunocastration as a practice has been referred to using different terms in a number of studies, such as “vaccine to prevent boar taint” [25], “medical castration with two injections” [21], or “immunovaccination by injecting a hormone like substance” [24]. It is often questioned whether the provided information may influence consumer attitudes. Several studies have already investigated this. Especially the possible effect of negative information is relevant, as the effect of a negative public campaign is often suggested as one of the main reasons that the pork production sector is reluctant to shift to the alternative of immunocastration.

The effect of terminology and extensiveness was studied by Heid and Hamm [22] using consumers of organically produced meat in Germany by providing three variants of information on castration and the alternatives: basic information, basic including pros and cons; and basic including pros and cons and the word hormone in the description of immunocastration. Basic versus extensive information did not result in a different ranking of immunocastration or castration without pain relief, but ranking of castration with anaesthesia improved while boar fattening moved one place downwards. Adding the term “hormone” to the explanation of immunocastration did not affect ranking, but more information did result in a stronger polarisation in attitudes. In another study, adding more information on immunocastration regarding welfare, quality, and practicability in the “pro” (reduction in animal pain and discomfort, absence of negative effects on meat quality, improved feed efficiency) and “con” (accidental self-injection risks for farm workers) sections did not affect Italian consumer attitudes in the study of Di Pasquale et al. [34]. Including benefits, including risks, or including benefits and risks did also not affect the acceptance of immunocastration in the study of Vanhonacker et al. [27], but the authors of this study note that the number of consumers per group may have been too low (*n* = 57) to draw clear conclusions. Adding information that IMMUNO may slightly increase the chance of boar taint shifted 9% of the consumers from totally acceptable to possibly acceptable in the study of Sodring et al. [28]. Besides the phrasing and extensiveness, the format of information may affect final outcome. Tuyttens et al. [35] studied the effect of extensiveness as well as format. Including more extensive information on paper did not affect consumer attitudes, whilst providing audiovisual information in addition to extensive information did increase the preference for immunocastration. In several studies, the authors did not report all the background information (explanations, pros and cons) in the publication that was provided to the participants, making it difficult to make comparisons between studies. Moreover, a good interpretation of the outcome of consumer studies is only possible when this information is included in the publication. Finally, the provided information needs to be thoroughly prepared and tested.

### 4.2. Evaluation of The Different Alternatives

Several studies indicate that consumers relate factors such as naturalness, i.e. space allowance and freedom (e.g., caged vs. group housed sows) more to animal welfare than surgical castration of piglets [21,23,27,36]. Unfamiliarity with pig production in general and this topic specifically can be one of the reasons for this. Within this study, we specifically focused on the impact of surgical castration to learn more about how and why consumers and stakeholders do or do not accept the different alternatives for surgical castration, although it is of course relevant to consider it in the broader context of pig production. These insights may help to increase implementation of the alternatives in terms of consumer and market acceptance, which is the main aim of this study. In order to better understand why consumers, accept or reject a certain option, respondents were asked to score 5 statements for each of the 4 options and to tick all terms that they related to each of the options as well as the ideal piece of pork. Based on the CATA terms that were presented to the respondents, the ideal piece of pork was characterized by “good quality”, “safe to eat”, “welfare friendly”, and “natural”. Out of the 4 presented options, ANAE aligned most with IDEAL. Overall, results of statements and CATA terms are in agreement, but the use of CATA terms is a fast way to gain additional insights into the consumer’s idea of an ideal piece of pork versus the alternatives. The study of Sodring [12] gave respondents an open question in which they could fill in why a certain option was considered unacceptable. The respondents indicated that animal welfare, food safety, and eating quality or a combination of these was a main driver for choosing a certain option. Within the present study, this is also reflected by the different axes found in the correspondence analysis with CONTROL separated from the alternatives and IDEAL due to animal welfare and a further partitioning between the alternatives based on quality and food safety.

In agreement with the scoring of statements and CATA scores, we found the highest acceptability (totally + possibly) for ANAE (85%), followed by IMMUNO (71%), and BOAR (49%) and the lowest acceptability for CONTROL (32%). The results of acceptability of the different options are highly consistent with the studies performed in Norway in 2008 and 2018 where acceptability was 89 and 88% for ANAE, 74 and 78% for IMMUNO, 32 and 38% for BOAR, and 15 and 22% for CONTROL [21,28]. In the study of Huber-Eicher [37], acceptance was also highest for ANAE (82%), followed by 60% acceptance for BOAR, and 53% for IMMUNO.

Whilst CONTROL is mainly considered unacceptable, the overall acceptance of ANAE is high, with few or no negative connotations. A successful elimination of the pain induced by surgical castration seems to be the crucial factor for consumer acceptance, regardless of the act of castration itself. Only a small share of respondents considered ANAE as unacceptable. In the study of Sodring [28], respondent comments in case of non-acceptance were “incomplete elimination of pain” (40%), “form of animal cruelty” (16%), “unnatural” (11%), and “unethical” (11%). A good and correct implementation of this practice in the field is therefore important to comply with consumer attitudes and expectations.

In this study, as well as in the study of Fredriksen et al. [21], consumers were generally either positive or neutral towards IMMUNO. This high acceptance rate can be explained by the trust that consumers have in their national food safety authority. Mancini et al. [38] also found that Italian consumers would accept immunocastration for pork, even for traditional products, if government institutions guarantee a strong involvement in quality, safety, and ethical treatment. Swedish consumers also showed no aversion towards immunocastration in the study of Viske et al. [23]. They indicated that animal welfare concerns outweighed biotechnology aversion or food safety risk when consumers compared immunocastration and surgical castration, while pork quality could be expected to be similar. Similar to our study, also Vanhonacker and Verbeke [25] found the highest preference for immunocastration in the animal welfare-oriented cluster. In general, consumers show a high confidence in the food safety of pork (72%). Besides the positive effect of immunocastration, trust in the food safety of pork probably further supports the respondents’ positive attitudes towards this alternative. A smaller part of the respondents did question the practice from the point of food safety, however. Similar observations were made by Mancini et al. [29]. The preference for a reduced use of drugs has been mentioned as drawback for this method [38], which may also be an issue for ANAE. Although the term hormone was not used in the infographic provided with our survey, frequency of the CATA term “hormone” was around 30% for IMMUNO and around 15% for the boars. The same observation was also made by Fredriksen et al. [20]. Several studies indicated that food safety (fear of drug residues in meat products, fear of unknown long-term effects and unnaturalness) can be a consumer concern and suggest the importance of guaranteeing product safety [21,25,28,29]. In future studies, it could be interesting to investigate if the term “hormone” in all cases has a negative connotation or not, i.e., as something natural vs. artificial. For BOAR, the word “hormone” as well as “natural” had a higher frequency. Further research into which information or assurance strategy could support the confidence of this consumer segment could be interesting. For example, in the study of Fredriksen et al. [21], acceptability did not improve when more information on food safety (no residuals, no risk for human safety) was added.

In general, consumers responded either neutrally or were against BOAR due to the chance of boar taint. Difference in acceptance or ranking of production of entire male pigs could be observed in other studies as well. Heid and Hamm [22] found that consumers of organic pork were willing to pay more for pork from boars compared to pork from piglets castrated without pain relief. However, these results are not supported by other studies. According to Viske and co-authors [23], Swedish consumers preferred pork from surgically castrated pigs to pork from boars, illustrating the weight of food quality in their decision making. Part of the explanation for this contrast could possibly be related to the different consumer groups (organic versus conventional). Heid and Hamm [22] also suggested a second explanation. They related their findings to the lack of familiarity with boar taint, as most participants responded that they did know this off-odour. The participants of the focus group study of Fredriksen et al. [20] nevertheless indicated that presence of boar taint or reduction of pork quality would reduce their pork consumption. Possibly, differences in the provided background information can also further explain the differences. A solution suggested by Heid and Hamm [22], namely providing samples with known levels of boar taint, could help to overcome this issue in case of focus groups or face-to-face interviews. The same attitude is seen in our study. Based on the responses in this questionnaire, respondents reported that they would address their complaints to the shop in person or via mail (40%), go to another shop to buy pork (33%), whilst 20% would just give it another chance. The success of acceptance of BOAR will therefore depend on (reduction of) the prevalence of boar taint, a good detection of tainted carcasses at the slaughter line, valorization of tainted carcasses, and the ability of a consumers to perceive boar taint in their home context.

Based on the study set-up of Fredriksen et al. [39], “possibly” and “don’t know” were also included as options to evaluate level of agreement with survey statements. In that study, the option of “possibly acceptable” was most used for ANAE (35%) and IMMUNO (26%), and the frequency of “don’t know” was highest for BOAR (40%). Within the current study, 29 to 35% of the respondents used “possibly acceptable” for each of the alternative options, whilst the option “‘don’t know” was less used for BOAR in our study (18%). “Difficult to understand” was included as one of the options as a CATA term, as it was expected to be related to these categories and was also suggested as relevant term during the pre-tests of this questionnaire. However, mainly CONTROL and IMMUNO had the highest frequency for “difficult to understand”, whilst this was lower for ANAE, indicating that this does not fully correspond with the selection of possibly acceptable and don’t know in the scale of acceptability. As suggested in other studies, a more neutral scoring may also indicate that respondents are feeling uncertain when asked to judge the different alternatives [40]. Based on the frequency of usage of these 2 categories in this study, it seems valuable to have these options included. Adding focus groups or open questions in addition to closed responses only could help to further increase insights in these attitudes.

### 4.3. Consumer and Stakeholder Segments

Making conclusions based on average results can be misleading when studying attitudes. Consumer attitudes can differ depending on (country-dependent) cultural background and habits, background knowledge and awareness, professional involvement, familiarity with agriculture, and socio-demographics. Differences in consumer and stakeholder characteristics were therefore explored in three clusters. A first cluster (45%) was against CONTROL and for ANAE and IMMUNO. The second cluster (38%) was against CONTROL and had a neutral attitude towards the other options. The third and smallest cluster (18%) has a neutral attitude towards CONTROL, is for ANAE, and against BOAR an IMMUNO.

These clusters reveal differences in the importance of the respondents’ attitude towards welfare, type of production system, and health and food safety, as discussed above, with the first cluster mainly welfare and taste oriented, the second low fat (health) and organic production, and the third mainly quality, safety, and local production. Moreover, country representation differs. In the first cluster, respondents of BEL, NOR, PRT, and SWE were represented more than average; in the second cluster, this was mainly DEU, and in the third, mainly BGR. Differences in acceptance per country often seem to be related to the current practice of either an alternative or CONTROL in each of the countries. Respondents of FRA and ESP accepted BOAR the most (>33%) and the respondents of BE and SW also showed a less negative attitude towards BOAR. Acceptance of ANAE was highest in SWE, NOR, PRT, and BGR. Indeed, in SWE as well as in NOR, ANAE is routine practice. In BGR, ANAE is also applied for home-grown pigs. Respondents from BEL, NOR, PRT, and SWE showed also more acceptance for the practice of IMMUNO, which is common but not routine in BEL, SWE, and NOR. For PRT, it is less clear why acceptance of ANAE and IMMUNO was relatively high, as it is not related to the current situation. A possible explanation is the higher share of female respondents in PRT. Representativity of the respondents can be an issue when interpreting these country-related results, and the necessary caution is surely needed. Larger scale studies are required to get more reliable insights in these country differences.

The clusters are also characterized by demographics (Table S1). Female respondents, who are most represented in cluster 1 and least in 3, generally assign more importance to animal welfare. Acceptability of CONTROL was indeed less for female than for male respondents and this is also reflected in the animal welfare related aspects, while acceptability of IMMUNO was higher. In this study, age differences between clusters were small, with younger respondents showing a slightly higher acceptance of IMMUNO, BOAR, and CONTROL. Previous studies indicated that the knowledge on the topic of surgical castration is lower in younger people and that acceptance of IMMUNO was higher at younger age (18–29 years) than for over-60-year-old respondents [21,28]. Cluster 2 showed fewest respondents with a university degree, which were characterized by an overall higher acceptance of each of the options. Living area did not strongly differ between clusters and had only little effect on the respondents’ attitudes.

More important to consider are the differences based on professional involvement and familiarity with agriculture. Professionally-involved respondents always gave a higher score to each of the statements for CONTROL, ANAE, and IMMUNO than people who were not professionally involved, while non-professionals indicated a slightly higher agreement for BOAR. Professional involvement vs. no involvement mainly increases the acceptance of CONTROL (48% vs. 27%) and IMMUNO (78 vs. 67%). However, also within the types of professional involvement, we can observe differences. Farmers showed the highest acceptability of CONTROL amongst the professions. They do consider ANAE and IMMUNO as more acceptable than CONTROL. However, within the professionally involved respondents, they are positive towards IMMUNO, but less than average, which is also the case for respondents involved in meat processing. The latter group was also least accepting BOAR and did not consider it as a good alternative, based on safety, welfare, as well as taste. Contrary to that, researchers are within the professional involved stakeholders most pro BOAR. Respondents professionally involved as butcher or in retail showed the lowest acceptance for CONTROL, mainly due to animal welfare. The shorter relationship of the butcher/retailer to the societal concern on animal welfare and maybe lower familiarity with agriculture may explain the difference in acceptance between butcher/retailer vs. farmer. The one alternative that all respondents were highly accepting was ANAE, and only veterinarians were slightly more not accepting this (10% vs. on average 5%).

Regarding the impact of familiarity with agriculture, trends are comparable to professional involvement. Respondents with no or limited familiarity with agriculture agree less with the statements related to CONTROL and ANAE. In line with the results for the farmers, respondents that grew up on a farm also had a lower acceptance of IMMUNO and BOAR compared to non-familiar stakeholders.

## 5. Conclusions

Large scale European consumer studies covering all alternatives are scarce and recent data on the acceptance of the main alternatives for surgical castration of piglets is lacking. Based on the current, exploratory study, it is not possible to draw far reaching conclusions for the average European consumers. A part of the respondents was also highly professionally involved and familiar with agriculture and had high awareness of piglet castration as we also wanted to evaluate stakeholders’ attitudes. Each of these aspects can influence the final percentages of acceptability; however, this may be the case in other studies as well. Conclusions are therefore applicable within these conditions.

Of the 4 presented options, the practice of CONTROL was overall least accepted (32%), whilst there is was high acceptance for ANAE (85%), followed by IMMUNO (71%), and BOAR (49%). The respondents strongly distinguished the current practice from the alternatives due to animal welfare reasons. This was even more outspoken by non-involved respondents, and also regional differences could be observed (i.e., with a higher acceptance of CONTROL in several Eastern European countries). Within the alternatives, further differentiation was made based on quality followed by safety, although a big group of correspondents choose against CONTROL without a preference for one alternative. For ANAE, there was a high acceptance and high consensus throughout the different respondent profiles, regardless of professional involvement or familiarity with agriculture, the clusters that could be defined, or the nationality of the respondent, as this option aligns well with expectations regarding welfare, safety, as well as good taste. A prerequisite is a practically and economically feasible, good, and correct implementation of this practice in the field to comply with attitudes and expectations for stakeholders as well as consumers. Also IMMUNO has a high degree of acceptance, although more profile related variation is visible. Overall, respondents have confidence in IMMUNO as practice, as it improves animal welfare and guarantees a good quality. The aspect of a possibly negative perception regarding food safety is often debated in the field. However, most respondents had a positive or neutral attitude and a high confidence in food safety in general. Based on the cluster analysis, only a small segment of the respondents (18%) was negative towards IMMUNO as well as to BOAR. When differentiation was made based on professional involvement and familiarity with agriculture, it could be observed that mainly farmers and processors show less acceptance for IMMUNO. More insights into these respondent groups specifically would therefore be interesting. How the respondents perceive the potential impact of boar taint will surely impact the acceptability of BOAR in attitude questionnaires. Within the proposed alternatives, the acceptability of BOAR was lower compared to the other two alternative options. Mainly professionally involved stakeholders and those involved in food processing specifically were less positive towards BOAR. Overall, a higher acceptance can be anticipated if the risk for boar taint can be limited by a reduction of boar taint, a reliable detection of tainted carcasses at the slaughter line, and valorization of tainted carcasses.

When discussing the implementation of an alternative to replace CONTROL, it should be economically and practically feasible for the farmer and accepted by the slaughterhouse, meat industry, butchers and retailers, as well as consumers. Based on this study, a moderate to very high acceptability can be expected from the alternatives, if performed according to best practices, whilst acceptability of CONTROL is clearly lower.

## Figures and Tables

**Table 1:** Demographic profile of the respondents by country and overall.

Country	BEL	BGR	HRV	CZE	DEU	ESP	FRA	ITA	NOR	POL	PRT	ROU	RUS	SRB	SWE	UKR	OVERALL
**N**	417	227	208	226	506	253	213	204	177	210	191	224	357	352	258	255	4278
**Gender ^1^**																	
Female	56	62	45	54	57	56	66	49	66	69	61	68	55	50	78	50	58
Male	43	37	55	44	43	42	33	51	33	30	37	27	43	46	21	46	40
**Age**																	
<25	4	25	12	13	10	10	12	4	1	63	25	37	24	18	16	36	18
25–39	38	27	27	36	27	40	27	36	29	16	30	42	41	47	41	26	34
40–64	48	42	60	47	42	47	49	56	63	18	41	21	33	32	38	35	41
>64	10	6	1	4	21	4	12	4	7	3	4	1	3	2	6	3	7
**Area**																	
Big city	15	67	47	36	30	18	25	12	24	42	25	41	72	42	26	33	35
Medium size city	30	25	23	20	22	57	31	38	28	15	50	22	24	24	34	29	29
Rural area/small town	55	8	30	44	48	25	45	50	49	43	25	37	5	34	40	38	36
**Education**																	
Primary/Secondary	20	10	13	17	45	8	8	7	6	29	2	20	20	22	8	9	18
Higher, non-university	33	4	9	4	25	10	24	19	20	14	1	9	26	11	5	21	16
University	47	86	78	79	30	82	69	75	75	57	97	71	54	67	87	71	66
**Professionally involved ^2^**																	
Yes	27	19	30	37	7	35	40	23	27	11	46	17	25	24	22	17	24
Supply	11	7	13	22	8	19	14	7	21	0	9	19	2	16	12	2	12
Farm	11	23	24	29	31	15	5	28	13	23	20	19	3	21	19	38	18
Veterinarian	16	74	14	11	25	38	14	33	15	68	25	16	11	13	51	17	24
Processing	11	14	21	2	8	3	6	7	8	0	8	32	41	38	0	14	14
Retail/butcher	22	12	2	1	11	0	1	2	2	9	5	16	15	12	0	0	7
Researcher	38	9	35	43	25	46	66	35	67	5	36	11	46	26	25	26	37
**Familiar with farming**																	
Regular non-professional contact	32	37	26	28	7	22	24	26	20	10	28	32	14	27	26	20	23
Grew up on a farm	16	23	26	16	11	10	16	15	15	18	22	16	7	20	13	47	17

^1^ Prefer not to answer this question was also included as possible answer option. ^2^ Multiple answers were possible. Participating countries are Belgium (BEL), Bulgaria (BGR), Czechia (CZE), Germany (DEU), Spain (ESP), France (FRA), Croatia (HRV), Italy (ITA), Norway (NOR), Poland (POL), Portugal (PRT), Romania (ROU), Russia (RUS), Serbia (SRB), Sweden (SWE) and Ukraine (UKR).

**Table 2:** Pork consumption characteristics, background knowledge, and experience of the respondents by country and overall.

Country	BEL	BGR	HRV	CZE	DEU	ESP	FRA	ITA	NOR	POL	PRT	ROU	RUS	SRB	SWE	UKR	OVERALL
**Pork consumption**																	
Less than once a week	16	10	8	11	20	14	19	23	25	15	16	18	21	13	35	26	18
1–2 times a week	31	33	38	28	46	34	43	40	45	35	34	37	33	33	34	37	36
3–4 times a week	35	30	34	36	20	36	27	30	18	28	35	30	28	30	22	23	29
More than 4 times a week	18	28	20	25	13	17	11	7	12	22	16	16	19	23	9	14	17
**Where do you buy your meat**																	
Butcher	54	67	61	65	45	55	43	56	2	18	40	44	39	47	8	52	44
Supermarket	81	49	57	73	80	62	69	51	98	75	78	51	57	46	92	31	66
Local producer	22	27	24	24	10	10	24	25	9	34	8	16	23	21	19	33	20
**Liking score ^1^**																	
Pork chops	5.1	6.0	5.8	6.2	5.8	5.5	5.7	5.7	5.8	5.5	6.0	5.1	6.0	5.1	5.1	6.1	5.6
Minced meat products	5.8	5.4	5.6	5.5	5.9	5.4	5.7	6.1	5.7	5.4	5.1	4.8	5.6	5.1	5.4	5.1	5.5
**Importance at purchase ^2^**																	
Lowest price	9	43	32	27	21	19	12	13	15	25	36	34	44	31	11	11	24
Good taste	84	95	95	80	82	88	91	89	90	83	85	93	89	92	83	66	86
Improved animal welfare	50	69	60	30	60	53	64	45	52	41	52	67	51	55	89	32	55
Produced locally	58	75	79	56	53	62	75	71	41	46	58	70	52	63	70	22	59
Produced organically	33	64	53	18	38	36	48	40	11	28	29	57	52	49	36	34	40
Low fat content	23	46	32	22	32	39	27	27	19	45	36	55	54	42	7	21	33
High tenderness	56	83	74	36	55	70	62	64	53	47	74	77	76	61	38	34	60
Easy to prepare	31	68	58	30	46	50	36	42	42	56	55	70	61	63	34	35	48
Guaranteed food safety	87	94	94	62	76	90	81	71	81	79	85	94	89	91	86	74	84
Low environmental impact	51	77	60	25	54	56	57	50	39	32	49	67	51	62	50	34	52
**Confident that meat is safe**	92	58	70	61	78	93	74	52	84	64	91	71	53	73	76	47	72
**Negative towards vaccination**	19	18	28	34	16	9	17	15	11	20	3	28	34	31	8	20	20
**Aware of piglet castration**	76	65	66	70	56	49	80	51	65	41	71	34	35	47	84	60	59
**Experience with bad smell/taste**	25	39	44	34	24	32	32	24	38	30	33	26	25	26	30	41	30
**Aware how mainly produced**	48	50	48	61	42	41	63	29	47	31	52	29	29	33	67	49	44
Production of boars ^3^	1	1	1	2	2	15	1	0	1	0	15	2	4	2	0	2	3
Immunocastration ^3^	9	0	2	2	6	5	1	2	7	3	4	0	5	1	7	5	4
With anaesthesia/analgesia ^3^	14	18	9	9	13	9	13	8	36	4	11	11	5	11	52	8	14
Without anaesthesia/analgesia ^3^	24	30	36	49	22	13	49	19	4	24	23	16	**14**	**19**	7	35	23

^1^ Scored on a 7 point-scale from strongly dislike to strongly like ^2^ Scored on a 7-point scale from not important at all to very important, % of scores 6 and 7 are presented ^3^ Per country, the value in bold is the main male pig production method and the underlined values are additionally applied methods.

**Table 3:** Effect of the demographic variables on the agreement to the different statements and the acceptability of the different options (Castration without pain relief—CONTROL, castration with anaesthesia—ANAE, Immunocastration—IMMUNO, and no castration—BOAR).

	WTB ^1^				SAFE ^1^				WELFARE ^1^				TASTE ^1^				ACCEPTANCE ^2^ % Acceptable/% Non-Acceptable			
	CONTROL	ANAE	IMMUNO	BOAR	CONTROL	ANAE	IMMUNO	BOAR	CONTROL	ANAE	IMMUNO	BOAR	CONTROL	ANAE	IMMUNO	BOAR	CONTROL	ANAE	IMMUNO	BOAR
**Gender**																				
Female	2.7 ^a^	5.3 ^a^	4.6 ^a^	3.5	4.8 ^a^	5.5 ^a^	4.6	4.5 ^a^	2.2 ^a^	5.1 ^b^	4.9 ^b^	4.7 ^a^	3.9 ^a^	5.1	4.4	3.1 ^a^	27/66	89/5	72/13	48/32
Male	3.3 ^b^	5.2 ^b^	4.4 ^b^	3.5	5.3 ^b^	5.6 ^b^	4.5	4.7 ^b^	2.7 ^b^	5.0 ^a^	4.7 ^a^	4.8 ^b^	4.4 ^b^	5.1	4.3	3.2 ^b^	38/55	89/6	69/17	51/34
**Age**																				
<25	3.2 ^b^	5.3	4.6 ^b^	3.5 ^ab^	4.7 ^b^	5.4 ^ab^	4.6	4.2 ^a^	2.6 ^b^	5.2 ^b^	4.9 ^c^	4.6 ^b^	4.2 ^c^	5.1	4.4 ^b^	3.3	38/55	91/3	73/10	49/32
25–39	2.9 ^a^	5.3	4.6 ^b^	3.6 ^b^	5.1 ^b^	5.6 ^ab^	4.6	4.7 ^b^	2.4 ^ab^	5.1 ^ab^	4.9 ^bc^	4.8 ^b^	4.2 ^bc^	5.1	4.4 ^ab^	3.1	33/61	88/7	72/14	52/31
40–64	2.9 ^a^	5.3	4.5 ^ab^	3.4 ^a^	5.1 ^b^	5.6 ^b^	4.6	4.7 ^b^	2.4 ^a^	5.1 ^ab^	4.8 ^ab^	4.8 ^b^	4.1 ^b^	5.2	4.4 ^ab^	3.0	31/63	89/6	70/16	49/34
>64	2.5 ^a^	4.9	4.1 ^a^	3.4 ^a^	4.1 ^a^	5.1 ^a^	4.2	4.1 ^a^	2.4 ^ab^	4.7 ^a^	4.3 ^a^	4.3 ^a^	3.4 ^a^	4.7	4.0 ^a^	3.3	22/70	86/6	63/17	33/38
**Education**																				
Primary/Secondary	2.8	4.9 ^a^	4.3 ^a^	3.6 ^ab^	4.1 ^a^	4.9 ^a^	4.1 ^a^	4.0 ^a^	2.6 ^b^	4.7 ^a^	4.4 ^a^	4.4 ^a^	3.5 ^a^	4.6 ^a^	4.1 ^a^	3.4 ^b^	24/65	83/8	62/15	44/30
Higher, non-university	2.8	5.1 ^a^	4.4 ^a^	3.8 ^b^	4.6 ^b^	5.3 ^b^	4.4 ^b^	4.5 ^b^	2.3 ^a^	4.9 ^a^	4.6 ^b^	4.9 ^b^	3.8 ^b^	4.9 ^b^	4.3 ^b^	3.5 ^b^	27/64	87/6	66/15	51/25
University	3.0	5.4 ^b^	4.7 ^b^	3.4 ^a^	5.3 ^c^	5.8 ^c^	4.7 ^c^	4.7 ^b^	2.4 ^a^	5.2 ^b^	5.0 ^c^	4.8 ^b^	4.4 ^c^	5.3 ^c^	4.5 ^b^	3.0 ^a^	35/59	91/5	74/14	50/35
**Profession ^3^**																				
No	2.7 ^a^	5.2 ^a^	4.5 ^a^	3.6 ^b^	4.7 ^a^	5.4 ^a^	4.4 ^a^	4.4 ^a^	2.3 ^a^	5.0 ^a^	4.7 ^a^	4.7	3.9 ^a^	5.0 ^a^	4.3 ^a^	3.3 ^b^	27/65	87/6	68/15	48/31
Yes	3.5 ^b^	5.6 ^b^	4.8 ^b^	3.2 ^a^	5.9 ^b^	6.1 ^b^	5.0 ^b^	4.9 ^b^	2.7 ^b^	5.3 ^b^	5.2 ^b^	4.8	4.9 ^b^	5.6 ^b^	4.7 ^b^	2.8 ^a^	48/48	92/5	78/13	51/40
Supply	3.8	5.7	4.8	3.2	5.9	6.0	4.9	5.0	3.2	5.2	5.1	4.8	5.0	5.5	4.8	3.0	52/43	90/7	77/12	50/37
Farm	4.1	5.5	4.3	2.9	5.6	5.8	4.5	4.4	3.6	5.3	4.8	4.2	5.0	5.4	4.3	2.8	61/33	92/4	70/19	43/49
Veterinarian	3.4	5.5	5.1	3.0	6.0	6.2	5.3	5.0	2.6	5.2	5.4	4.6	4.8	5.6	4.8	2.6	50/48	89/10	86/11	49/47
Processing	3.7	5.4	4.2	2.5	5.3	5.9	4.5	3.9	3.1	5.6	4.8	3.9	4.8	5.7	4.4	2.4	47/46	92/4	70/17	29/61
Retail/butcher	3.2	5.0	4.8	2.7	5.2	5.6	4.9	4.1	2.6	4.9	5.0	4.3	4.3	5.1	4.5	2.8	38/60	95/5	77/11	43/46
Researcher	3.4	5.8	4.9	3.6	6.2	6.3	5.2	5.5	2.5	5.3	5.3	5.2	5.2	5.7	4.9	2.8	46/52	95/4	82/12	63/30
**Familiarity**																				
No	2.5 ^a^	5.1 ^a^	4.6 ^c^	3.7 ^c^	4.7 ^a^	5.4 ^a^	4.6 ^b^	4.6 ^b^	2.2 ^a^	4.9 ^a^	4.8 ^b^	4.8 ^b^	3.8 ^a^	4.9 ^a^	4.4 ^b^	3.3 ^c^	24/69	87/6	73/12	52/28
Contact	3.3 ^b^	5.5 ^b^	4.5 ^b^	3.4 ^b^	5.4 ^b^	5.8 ^b^	4.6 ^b^	4.7 ^b^	2.6 ^b^	5.3 ^b^	4.9 ^ab^	4.9 ^b^	4.5 ^b^	5.4 ^b^	4.4 ^ab^	3.0 ^b^	40/53	90/5	68/17	49/37
Farm	3.8 ^c^	5.5 ^b^	4.3 ^a^	3.0 ^a^	5.4 ^b^	5.7 ^b^	4.4 ^a^	4.2 ^a^	3.0 ^c^	5.3 ^b^	4.7 ^a^	4.4 ^a^	4.7 ^b^	5.3 ^b^	4.2 ^a^	2.7 ^a^	50/44	91/5	65/20	40/46
OVERALL	2.9	5.3	4.5	3.5	5.0	5.5	4.6	4.6	2.4	5.1	4.8	4.7	4.1	5.1	4.4	3.1	32/61	89/6	71/14	49/33
RMSE	1.7	1.9	2.0	2.0	1.8	2.1	1.8	1.8	2.1	2.0	1.6	1.7	1.9	1.7	1.9	2.0				

^a,b^ different superscripts indicate significant differences within demographic variables per option (*p* < 0.05) ^1^ Statements were scored on a scale from 1 (totally disagree) to 7 (totally agree) “If the castration method was clearly labelled, I would surely buy…” (*WTB*), “I think that is totally safe to eat meat from …” (*SAFE*), “When I think about the following production method, it seems logical to me that animal welfare is best for …” (*WELFARE*), “If I consider the tastiness of meat, I prefer meat from…” (*TASTE*) ^2^ Acceptability was scored as totally acceptable, possibly acceptable, not acceptable or don’t know. Acceptable is the sum of totally acceptable and possibly acceptable). ^3^ Statistical analysis between professionally involved versus not involved.

**Table 4:** Comparison of the statements and CATA scoring over clusters for all respondents and all non-professionally involved respondents of the different options (Castration without pain relief—CONTROL, castration with anaesthesia—ANAE, Immunocastration—IMMUNO, and no castration—BOAR).

		Cluster 1	Cluster 2	Cluster 3	RMSE
**N (%)**		1910 (45%)	1619 (38%)	749 (18%)	
**Statements ^1^**					
WTB	CONTROL	2.8 ^a^	2.8 ^a^	3.6 ^b^	1.5
	ANAE	5.6 ^b^	4.3 ^a^	6.3 ^c^	1.5
	IMMUNO	5.8 ^c^	3.8 ^b^	2.9 ^a^	2.0
	BOAR	3.5 ^b^	3.9 ^c^	2.8 ^a^	1.9
SAFE	CONTROL	5.5 ^a^	3.9 ^b^	5.9 ^c^	1.3
	ANAE	6.2 ^a^	4.3 ^b^	6.5 ^c^	1.4
	IMMUNO	5.9 ^a^	3.7 ^b^	2.9 ^c^	2.1
	BOAR	5.1^a^	4.1^b^	4.1 ^b^	1.9
WELFARE	CONTROL	2.3 ^a^	2.5 ^ab^	2.6 ^b^	1.6
	ANAE	5.4 ^a^	4.2 ^b^	6.3 ^c^	1.5
	IMMUNO	6.0 ^a^	3.9 ^b^	3.8 ^b^	2.1
	BOAR	4.9 ^a^	4.6 ^b^	4.6 ^b^	2.0
TASTE	CONTROL	4.3 ^a^	3.6 ^b^	4.9 ^c^	1.4
	ANAE	5.5 ^a^	4.0 ^b^	6.2 ^c^	1.4
	IMMUNO	5.5 ^a^	3.6 ^b^	3.1 ^c^	1.8
	BOAR	3.0 ^a^	3.7 ^b^	2.3 ^c^	2.0
**CATA (%)**					
Safe to eat	CONTROL	47 ^a^	32 ^b^	60 ^c^	48
	ANAE	62 ^a^	36 ^b^	69 ^c^	44
	IMMUNO	49 ^a^	21 ^b^	11 ^c^	48
	BOAR	40 ^a^	29 ^b^	36 ^a^	17
Hormones	CONTROL	4	4	3	21
	ANAE	3 ^a^	8 ^b^	2 ^a^	45
	IMMUNO	23 ^a^	30 ^b^	52 ^c^	33
	BOAR	14 ^a^	9 ^b^	20 ^c^	50
Animal welfare friendly	CONTROL	7 ^a^	10 ^b^	9 ^ab^	49
	ANAE	52 ^a^	34 ^b^	66 ^c^	46
	IMMUNO	61 ^a^	24 ^b^	32 ^c^	50
	BOAR	46 ^a^	41 ^b^	45 ^ab^	11
Natural	CONTROL	21 ^a^	25 ^b^	37 ^c^	41
	ANAE	21 ^a^	18 ^a^	33 ^b^	33
	IMMUNO	16 ^a^	13 ^b^	5 ^c^	50
	BOAR	51 ^a^	45 ^b^	50 ^ab^	35
Good quality	CONTROL	45 ^a^	35 ^b^	61 ^c^	47
	ANAE	67 ^a^	41 ^b^	78 ^c^	45
	IMMUNO	59 ^a^	25 ^b^	20 ^c^	41
	BOAR	22 ^a^	24 ^a^	14 ^b^	12
Bad taste	CONTROL	5 ^a^	7 ^b^	5 ^a^	15
	ANAE	1 ^a^	4 ^b^	1 ^a^	23
	IMMUNO	2 ^a^	7 ^b^	13 ^c^	48
	BOAR	61 ^a^	37 ^b^	75 ^c^	48

^a–c^: different superscripts indicate significant differences within clusters per option (*p* < 0.05) ^1^ Statements were scored on a scale from 1 (totally disagree) to 7 (totally agree) “If the castration method was clearly labelled, I would surely buy…” (*WTB*), “I think that is totally safe to eat meat from …” (*SAFE*), “When I think about the following production method, it seems logical to me that animal welfare is best for …” (*WELFARE*), “If I consider the tastiness of meat, I prefer meat from…” (*TASTE*).

## Supplementary Materials

The following are available online at https://www.mdpi.com/2076-2615/10/10/1758/s1, Figure S1: Acceptability of castration alternatives per type of professionally involved stakeholder for (a) Castration without pain relief—CONTROL, (b) castration with anaesthesia—ANAE, (c) Immunocastration—IMMUNO, (d) no castration—BOAR, Figure S2: Acceptability of castration alternatives per cluster for (a) Castration without pain relief—CONTROL, (b) castration with anaesthesia—ANAE, (c) Immunocastration—IMMUNO, (d) no castration—BOAR, Table S1: Overview of demographics per cluster for all respondents and all not professionally involved respondents.
