# Erratum: Aluwé, M. et al. Exploratory Survey on European Consumer and Stakeholder Attitudes towards Alternatives for Surgical Castration of Piglets. *Animals* 2020, *10*, 1758

**DOI:** 10.3390/ani10122388

**Published:** 2020-12-14

**Authors:** Marijke Aluwé, Evert Heyrman, João M. Almeida, Jakub Babol, Gianni Battacone, Jaroslav Čítek, Maria Font i Furnols, Andriy Getya, Danijel Karolyi, Eliza Kostyra, Kevin Kress, Goran Kušec, Daniel Mörlein, Anastasia Semenova, Martin Škrlep, Todor Stoyanchev, Igor Tomašević, Liliana Tudoreanu, Maren Van Son, Sylwia Żakowska-Biemans, Galia Zamaratskaia, Alice Van den Broeke, Macarena Egea

**Affiliations:** 1 Animal Sciences Unit, Flanders Research Institute for Agriculture, Fisheries and Food (ILVO), 9090 Melle, Belgium; evert.heyrman@ilvo.vlaanderen.be (E.H.); alice.vandenbroeke@ilvo.vlaanderen.be (A.V.d.B.); 2 Instituto Nacional de Investigação Agrária e Veterinária (INIAV), Quinta da Fonte Boa, 2005-048 Vale de Santarém, Portugal; joaoalmeida@iniav.pt; 3 Department of Biomedical Science and Veterinary Public Health, Swedish University of Agricultural Sciences, P.O. Box 7015, 750 07 Uppsala, Sweden; jakub.babol@slu.se; 4 Dipartimento di Agraria, Università Degli Studi di Sassari, Viale Italia 39, 07100 Sassari, Italy; battacon@uniss.it; 5 Department of Animal Science, Faculty of Agrobiology, Food and Natural Resources, Czech University of Life Sciences Prague (CZU), Kamycka 129, 16500 Prague, Czech Republic; citek@af.czu.cz; 6 Institute for Food and Agriculture Research and Technology (IRTA), Product Quality Program, Finca Camps i Armet, 17121 Girona, Spain; maria.font@irta.cat; 7 Animal Breeding Department, National University of Life and Environmental Sciences of Ukraine (NULES), Henerala Rodimtseva 19, 03041 Kyiv, Ukraine; getya@ukr.net; 8 Department of Animal Science, Faculty of Agriculture (UNIZG), University of Zagreb, Svetosimunska Cesta 25, 10 000 Zagreb, Croatia; dkarolyi@agr.hr; 9 Institute of Human Nutrition Sciences, Warsaw University of Life Sciences (WULS-SGGW), ul. Nowoursynowska 159c, 02-787 Warsaw, Poland; eliza_kostyra@sggw.edu.pl (E.K.); sylwia_zakowska_biemans@sggw.edu.pl (S.Ż.-B.); 10 Department of Behavioral Physiology of Livestock, Institute of Animal Science, University of Hohenheim, Garbenstraße 17, 70599 Stuttgart, Germany; kress.kevin@uni-hohenheim.de; 11 Department of Animal Production and Biotechnology, Faculty of Agrobiotechnical Sciences Osijek, University of Osijek, Vladimira Preloga 1, 31000 Osijek, Croatia; gkusec@fazos.hr; 12 Department of Animal Sciences, University of Göttingen, Albrecht-Thaer-Weg 3, 37075 Göttingen, Germany; daniel.moerlein@uni-goettingen.de; 13 V.M. Gorbatov Federal Research Center for Food Systems of Russian Academy of Sciences, 26, Talalikhina str., 109316 Moscow, Russia; a.semenova@fncps.ru; 14 Agricultural Institute of Slovenia, Hacquetova Ulica 17, SI-1000 Ljubljana, Slovenia; martin.skrlep@kis.si; 15 Department of Food Safety and Control of Foodstuffs Animal Origin, Faculty of Veterinary Medicine, Trakia University, Students Campus, 6000 Stara Zagora, Bulgaria; todor.stoyanchev@uni-sz.bg; 16 Department of Animal Source Food Technology, Faculty of Agriculture, University of Belgrade, Nemanjina 6, 11080 Belgrade, Serbia; tbigor@agrif.bg.ac.rs; 17 Interdisciplinary Laboratory for Research on Heavy Metals Accumulation in the Food Chain and Modeling, University of Agronomic Sciences and Veterinary Medicine, Faculty of Veterinary Medicine, 011464 Bucharest, Romania; liliana_tudoreanu223@hotmail.co.uk; 18 Norsvin SA, Storhamargata 44, 2317 Hamar, Norway; maren.van.son@norsvin.no; 19 Department of Molecular Sciences, Swedish University of Agricultural Sciences, P.O. Box 7015, 750 07 Uppsala, Sweden; Galia.zamaratskaia@slu.se; 20 Department of Food Science and Technology, Veterinary Faculty, University of Murcia, 30071 Murcia, Spain; macarena.egea@um.es

The authors wish to make the following corrections to this paper [1]:

## 1. Change in Acknowledgements

This publication was funded by COST Action “Innovative approaches in pork production with entire males”-IPEMA (CA 15215), supported by COST (European Cooperation in Science and Technology). The authors would like to thank Miriam Levenson (ILVO) and all people listed for helping with the pre-testing, translation or distribution of the questionnaire: L. Paternoster (BE), M. Frijlinck (BE), M. Lourenco (BE), O. Moreira (PT), J. Prates (PT), M. Bonneau (FR), P. Lawlor (IR), L. Doran (UK), J.E. Haugen (NO), M. Candek-Potokar (SL), M. Verhaagh (DE), D. Nakov (MK), M. Veneziani (IT) and many more colleagues and members of the Cost IPEMA network. In sweet memory and honor of Ulrike Weiler.
