# Supplementary material for: Exploratory Survey on European Consumer and Stakeholder Attitudes towards Alternatives for Surgical Castration of Piglets
Source: Animals (Basel). 2020 Sep 28;10(10):1758. doi: 10.3390/ani10101758 (PMC7600221; doi:10.3390/ani10101758)
Supplement: Supplementary file 1 [file animals-10-01758-s001.zip › animals-916288-supplementary.docx]

Article

Exploratory Survey on European Consumer and Stakeholder Attitudes towards Alternatives for Surgical Castration of Piglets

Marijke Aluwé ^1^, Evert Heyrman ^1^, João M. Almeida ^2^, Jakub Babol ^3^, Gianni Battacone ^4^, Jaroslav Čitek ^5^, Maria Font i Furnols ^6^, Andriy Getya ^7^, Danijel Karolyi ^8^, Eliza Kostyra ^9^, Kevin Kress ^10^, Goran Kušec ^11^, Daniel Mörlein ^12^, Anastasia Semenova ^13^, Martin Škrlep^14^, Todor Stoyanchev ^15^, Igor Tomašević ^16^, Liliana Tudoreanu ^17^, Maren Van Son ^18^, Sylwia Żakowska -Biemans ^9^, Galia Zamaratskaia ^19^, Alice Van den Broeke ^1^ and Macarena Egea ^20^

^1^ Flanders Research Institute for Agriculture, Fisheries and Food (ILVO), Animal Sciences Unit, 9090 Melle, Belgium; marijke.aluwe@ilvo.vlaanderen.be (M.A.), evert.heyrman@ilvo.vlaanderen.be (E.H.), alice.vandenbroeke@ilvo.vlaanderen.be (A.V.d.B.)

^2^ Instituto Nacional de Investigação Agrária e Veterinária (INIAV), Quinta da Fonte Boa, 2005-048 Vale de Santarém, Portugal; joaoalmeida@iniav.pt

^3^ Department of Biomedical Science and Veterinary Public Health, Swedish University of Agricultural Sciences, Box 7015, 750 07 Uppsala, Sweden; jakub.babol@slu.se

^4^ Dipartimento di Agraria, Università degli Studi di Sassari, Viale Italia 39, 07100 Sassari, Italy; battacon@uniss.it

^5^ Department of Animal Science, Faculty of Agrobiology, Food and Natural Resources, Czech University of Life Sciences Prague (CZU), Kamycka 129, 16500 Prague, Czech Republic; citek@af.czu.cz

^6^ Institute for Food and Agriculture Research and Technology (IRTA), Product Quality Program, Finca Camps i Armet, 17121 Monells, Girona, Spain; maria.font@irta.cat

^7^ Animal Breeding Department, National University of Life and Environmental Sciences of Ukraine (NULES), Henerala Rodimtseva 19, 03041 Kyiv, Ukraine; getya@ukr.net

^8^ Department of Animal Science, Faculty of Agriculture (UNIZG), University of Zagreb, Svetosimunska cesta 25, 10 000 Zagreb, Croatia; dkarolyi@agr.hr

^9^ Institute of Human Nutrition Sciences, Warsaw University of Life Sciences (WULS-SGGW), ul. Nowoursynowska 159c, 02-787 Warsaw, Poland; eliza_kostyra@sggw.edu.pl (E.K.), sylwia_zakowska_biemans@sggw.edu.pl (S.Ż.-B.)

^10^ Department of Behavioral Physiology of Livestock, Institute of Animal Science, University of Hohenheim, Garbenstraße 17, 70599 Stuttgart, Germany; kress.kevin@uni-hohenheim.de

^11^ Department of Animal Production and Biotechnology, Faculty of Agrobiotechnical Sciences Osijek, University of Osijek, Vladimira Preloga 1, 31000, Osijek, Croatia; gkusec@fazos.hr

^12^ Department of Animal Sciences, University of Göttingen, Albrecht-Thaer-Weg 3, 37075 Göttingen, Germany; daniel.moerlein@uni-goettingen.de

^13^ V.M. Gorbatov Federal Research Center for Food Systems of Russian Academy of Sciences; 26, Talalikhina str., Moscow, 109316, Russian Federation; a.semenova@fncps.ru

^14^ Agricultural Institute of Slovenia, Hacquetova ulica 17, SI-1000 Ljubljana, Slovenia; martin.skrlep@kis.

^15^ Department of Food safety and control of foodstuffs animal origin, Faculty of Veterinary medicine, Trakia University, Students campus 6000, Stara Zagora, Bulgaria; todor.stoyanchev@uni-sz.bg

^16^ Department of Animal Source Food Technology, Faculty of Agriculture, University of Belgrade, Nemanjina 6, 11080 Belgrade, Serbia; tbigor@agrif.bg.ac.rs

^17^ Interdisciplinary Laboratory for Research on Heavy Metals Accumulation in the Food Chain and Modeling, University of Agronomic Sciences and Veterinary Medicine, Faculty of Veterinary Medicine, Bucharest, Romania; liliana_tudoreanu223@hotmail.co.uk

^18^ Norsvin SA, Storhamargata 44, 2317 Hamar, Norway; maren.van.son@norsvin.no

^19^ Department of Molecular Sciences, Swedish University of Agricultural Sciences, Box 7015, 750 07 Uppsala, Sweden; Galia.zamaratskaia@slu.se

^20^ Department of Food Science and Technology, Veterinary Faculty, University of Murcia, 30071, Espinardo Murcia, Spain; macarena.egea@um.es

***** Correspondence: marijke.aluwe@ilvo.vlaanderen.be

Received: 14 August 2020; Accepted: 16 September 2020; Published: date


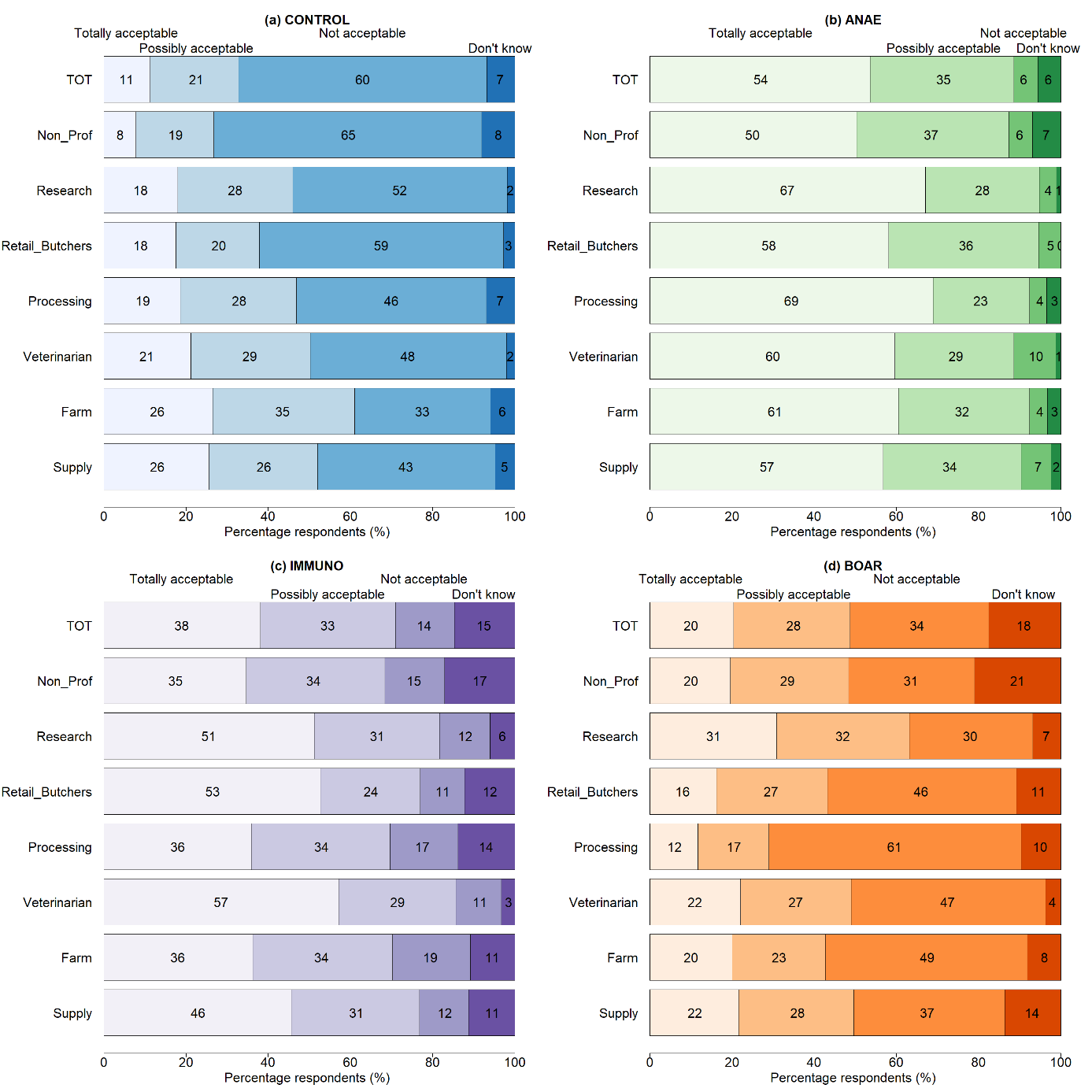


**Figure S1.** Acceptability of castration alternatives per type of professionally involved stakeholder for (a) Castration without pain relief—CONTROL, (b) castration with anaesthesia—ANAE, (c) Immunocastration—IMMUNO, (d) no castration—BOAR.


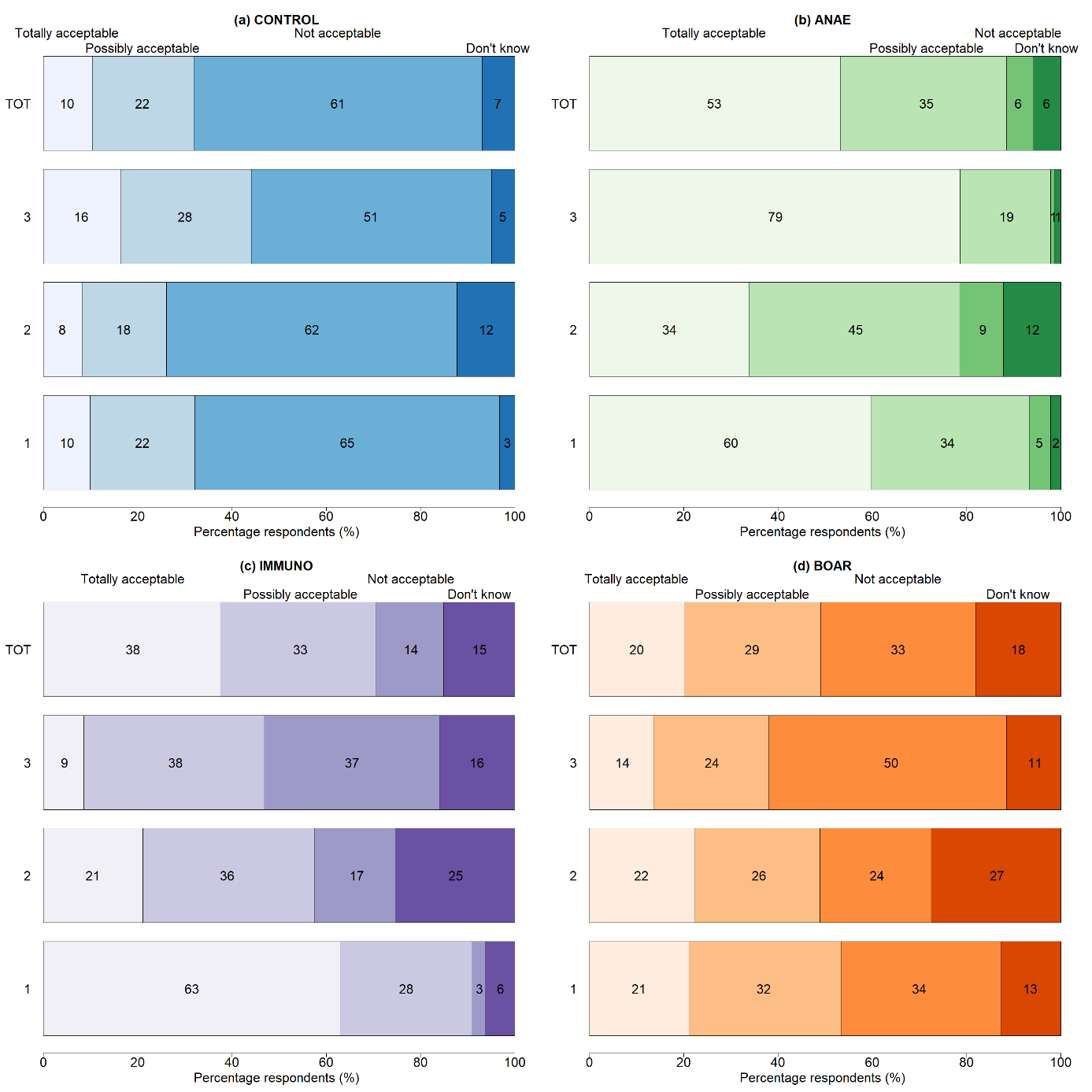


**Figure S2.** Acceptability of castration alternatives per cluster for (a) Castration without pain relief—CONTROL, (b) castration with anaesthesia—ANAE, (c) Immunocastration—IMMUNO, (d) no castration—BOAR*.*

**Table S1.** Overview of socio-demographics per cluster for all respondents and all not professionally involved respondents (in %).

|  |  | **All Respondents** | | |
| --- | --- | --- | --- | --- |
|  |  | **C1** | **C2** | **C3** |
| N |  | 1910 | 1619 | 749 |
| % |  | 45 | 38 | 18 |
| Country | BEL | 61 | 24 | 15 |
|  | BGR | 35 | 25 | 40 |
|  | HRV | 40 | 37 | 23 |
|  | CZE | 38 | 36 | 26 |
|  | DEU | 34 | 61 | 6 |
|  | ESP | 53 | 35 | 12 |
|  | FRA | 48 | 30 | 22 |
|  | ITA | 35 | 49 | 16 |
|  | NOR | 62 | 21 | 17 |
|  | POL | 41 | 42 | 17 |
|  | PRT | 63 | 22 | 15 |
|  | ROU | 35 | 49 | 16 |
|  | RUS | 39 | 38 | 23 |
|  | SRB | 30 | 50 | 19 |
|  | SWE | 77 | 14 | 9 |
|  | UKR | 35 | 46 | 19 |
| Gender | Female | 60 | 58 | 54 |
|  | Male | 38 | 39 | 44 |
| Age | <25 | 17 | 20 | 16 |
|  | 25–39 | 35 | 33 | 34 |
|  | 40–64 | 43 | 39 | 44 |
|  | >64 | 5 | 8 | 6 |
| Education | Primary + secondary | 12 | 23 | 12 |
|  | Non-university | 14 | 19 | 16 |
|  | University | 73 | 56 | 70 |
| Professionally involved | No | 69 | 87 | 73 |
| Familiar with agriculture | No | 59 | 68 | 46 |
|  | Regular contact | 24 | 18 | 30 |
|  | Grew up on farm | 17 | 14 | 25 |
| Attitude towards vaccines | Positive | 88 | 73 | 73 |
| Confidence in food safety | Yes | 79 | 63 | 71 |
| Aware of piglet castration | No | 31 | 55 | 36 |
| Bad experience | Yes | 32 | 27 | 32 |
| Pork consumption | <1 | 18 | 21 | 14 |
|  | 1–2 | 36 | 39 | 33 |
|  | 3–4 | 29 | 27 | 31 |
|  | >4 | 12 | 10 | 16 |
|  | Daily | 5 | 4 | 6 |
| Purchase attributes 1 | Lowest price | 23 | 23 | 27 |
|  | Good taste | 88 | 82 | 90 |
|  | Animal welfare | 58 | 53 | 52 |
|  | Produced locally | 59 | 56 | 65 |
|  | Produced organically | 36 | 44 | 42 |
|  | Low fat content | 30 | 36 | 35 |
|  | High tenderness | 63 | 55 | 63 |
|  | Easy to prepare | 48 | 47 | 50 |
|  | Food safety | 85 | 80 | 88 |
|  | Environmental impact | 51 | 51 | 53 |

^1^ Scored on a 7-point scale from not important at all to very important, % of scores 6 and 7 are presented.

| 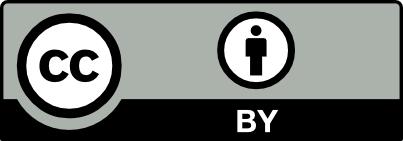 | © 2020 by the authors. Submitted for possible open access publication under the terms and conditions of the Creative Commons Attribution (CC BY) license (http://creativecommons.org/licenses/by/4.0/). |
| --- | --- |
